# Diel rewiring and positive selection of ancient plant proteins enabled evolution of CAM photosynthesis in *Agave*

**DOI:** 10.1186/s12864-018-4964-7

**Published:** 2018-08-06

**Authors:** Hengfu Yin, Hao-Bo Guo, David J. Weston, Anne M. Borland, Priya Ranjan, Paul E. Abraham, Sara S. Jawdy, James Wachira, Gerald A. Tuskan, Timothy J. Tschaplinski, Stan D. Wullschleger, Hong Guo, Robert L. Hettich, Stephen M. Gross, Zhong Wang, Axel Visel, Xiaohan Yang

**Affiliations:** 1 Biosciences Division, Oak Ridge National Laboratory, Oak Ridge, TN 37831 USA; 2 Department of Biology, University of Tennessee, Knoxville, TN 37996 USA; 3 School of Natural and Environmental Sciences, Newcastle University, Newcastle upon Tyne, NE1 7RU UK; 4 DOE-Center for Bioenergy Innovation (CBI), Oak Ridge National Laboratory, Oak Ridge, TN 37831 USA; 5 Chemical Sciences Division, Oak Ridge National Laboratory, 37831, Oak Ridge, TN USA; 6 Department of Biology, Morgan State University, Baltimore, MD 21251 USA; 7 Environmental Sciences Division, Oak Ridge National Laboratory, Oak Ridge, TN 37831 USA; 8 DOE Joint Genome Institute, Walnut Creek, CA 94598 USA; 9 School of Natural Sciences, University of California, Merced, CA 95343 USA; 10 Environmental Genomics and Systems Biology Division, Lawrence Berkeley National Laboratory, Berkeley, CA 94720 USA; 11 Present address: Research Institute of Subtropical Forestry, Chinese Academy of Forestry, Zhejiang, 311400 Hangzhou China; 12 Present address: Illumina, Inc., San Diego, CA 92122 USA

**Keywords:** Crassulacean acid metabolism, Photosynthesis, Comparative genomics, Transcriptome, Positive selection, Circadian rhythm

## Abstract

**Background:**

Crassulacean acid metabolism (CAM) enhances plant water-use efficiency through an inverse day/night pattern of stomatal closure/opening that facilitates nocturnal CO_2_ uptake. CAM has evolved independently in over 35 plant lineages, accounting for ~ 6% of all higher plants. *Agave* species are highly heat- and drought-tolerant, and have been domesticated as model CAM crops for beverage, fiber, and biofuel production in semi-arid and arid regions. However, the genomic basis of evolutionary innovation of CAM in genus *Agave* is largely unknown.

**Results:**

Using an approach that integrated genomics, gene co-expression networks, comparative genomics and protein structure analyses, we investigated the molecular evolution of CAM as exemplified in *Agave*. Comparative genomics analyses among C_3_, C_4_ and CAM species revealed that core metabolic components required for CAM have ancient genomic origins traceable to non-vascular plants while regulatory proteins required for diel re-programming of metabolism have a more recent origin shared among C_3_, C_4_ and CAM species. We showed that accelerated evolution of key functional domains in proteins responsible for primary metabolism and signaling, together with a diel re-programming of the transcription of genes involved in carbon fixation, carbohydrate processing, redox homeostasis, and circadian control is required for the evolution of CAM in *Agave*. Furthermore, we highlighted the potential candidates contributing to the adaptation of CAM functional modules.

**Conclusions:**

This work provides evidence of adaptive evolution of CAM related pathways. We showed that the core metabolic components required for CAM are shared by non-vascular plants, but regulatory proteins involved in re-reprogramming of carbon fixation and metabolite transportation appeared more recently. We propose that the accelerated evolution of key proteins together with a diel re-programming of gene expression were required for CAM evolution from C_3_ ancestors in *Agave*.

**Electronic supplementary material:**

The online version of this article (10.1186/s12864-018-4964-7) contains supplementary material, which is available to authorized users.

## Background

Among the three modes of photosynthesis in higher plants, the C_3_ pathway is the most ancient and common, occurring in approximately 90% of higher plant species. C_4_ and CAM photosynthesis, which account for approximately 3 and 6% of higher plant species, respectively, are evolutionarily derived from C_3_ photosynthesis and are believed to have arisen in response to selective pressures imposed by global reductions in atmospheric CO_2_ concentration (C_4_) and water limitation (CAM) [1, 2]. Both C_4_ and CAM plants capture CO_2_ via an initial carboxylation reaction catalyzed outside the chloroplast by phosphoenolpyruvate carboxylase (PEPC), which then subsequently delivers the captured CO_2_ at increased concentration to ribulose-1,5-bisphosphate carboxylase/oxygenase (RuBisCO) in the chloroplast. While C_4_ operates via a spatial separation of carboxylases in different cell types, CAM operates via a temporal day/night separation of RuBisCO and PEPC with net CO_2_ uptake shifted predominantly to the night. Nocturnal CO_2_ uptake is accompanied by an inverse (compared to C_3_ and C_4_) day/night pattern of stomatal closure/opening in CAM that results in improved water-use efficiency (i.e., CO_2_ fixed per molecule of H_2_O lost) that is six-fold higher than C_3_ plants and 3-fold higher than C_4_ plants under comparable conditions [3].

Circadian regulation of gene expression has been implicated as a core component in the diel re-programming of metabolism that distinguishes CAM from C_3_ and C_4_ photosynthesis [4, 5]. For instance, the nocturnal activation of PEPC via phosphorylation in CAM plants is catalysed by a dedicated PEPC kinase (PPCK), the transcript abundance of which is regulated by the circadian clock [5, 6]. A number of clock genes which were examined in the facultative CAM species *Mesembryanthemum crystallinum* indicated both conserved and divergent functions of genes encoding components of the core oscillator and the clock output pathways in this species [7]. In *Opuntia ficus-indica* (a constitutive CAM species), a transcriptomics study identified several genes implicated in the CAM biochemical pathway and in the circadian clock that displayed a unique 12-h periodicity [8] different from C_3_ species like *Arabidopsis*. Other work has suggested that the rhythmic expression of genes required for CAM is a consequence of diel changes in CAM-defining metabolites, such as malic acid [3]. Together, these studies suggest that modifications to the circadian clock, including both input and output pathways, might be critical to CAM evolution. Still, how the circadian clock was integrated within signalling and core biochemical components of CAM during the evolution of this pathway remains unestablished. Genome-wide gene expression profiling in model species has greatly facilitated our understanding of gene regulatory networks that are relevant to circadian regulation [9]. In this work, we develop a genome-wide approach to investigate the evolution of the core metabolic and regulatory elements of CAM via cross-species comparisons.

CAM and C_4_ photosynthesis are thought to have evolved from C_3_ ancestors multiple times in response to limitations in CO_2_ and water. It has been proposed that a propensity for frequent mutation or selection of certain regulatory genes during the early stages of evolution underpinned adaptations to environmental stress conditions [1]. Several studies in C_4_ have uncovered positive selection of key components of photosynthesis [10, 11], despite the conserved nature of many of these genes. For example, enzymatic kinetics and structure modelling of positively-selected residues of RuBisCO subunits in *Flaveria* indicated that these key amino acid substitutions were relevant to the functional diversification of C_4_ [11]. Although little has been characterized in CAM plants, the systematic analysis of positive selection in genes implicated in the circadian clock, photosynthesis, and CAM biochemistry could provide insight to unlock the mechanism(s) underlying CAM evolution.

*Agave* species are constitutive CAM species and many are important economic crops for beverage and fiber production [12, 13]. The water conserving properties of CAM have also highlighted the value of *Agave* as potential dedicated bioenergy feedstocks in semi-arid regions [14, 15]. Hence, a fundamental understanding of regulatory pathways underlying CAM in *Agave* is critical for efforts directed at engineering this pathway into C_3_ crops to improve water-use efficiency [13, 16]. All of the enzymes required for C_4_ and CAM appear to be homologs of ancestral forms found in C_3_ species [17], yet the specific genomic origins and genetic regulation of diel reprogramming of metabolism that distinguishes CAM from C_3_ and C_4_ photosynthesis are largely unknown [18]. In this study, we investigated the molecular evolution of CAM in *Agave* using an approach that integrated gene co-expression networks, comparative genomics, and protein structure analysis. Our findings demonstrate that 1) core metabolic components required for CAM have ancient genomic origins traceable to non-vascular plants, 2) regulatory proteins required for diel re-programming of metabolism have a more recent origin shared among C_3_, C_4_, and CAM species and 3) accelerated evolution of key proteins together with a diel re-programming of gene expression were required for the evolution of CAM in *Agave*.

## Results

### CAM physiology and identification of co-expression modules relevant to CAM

**Fig. 1:**
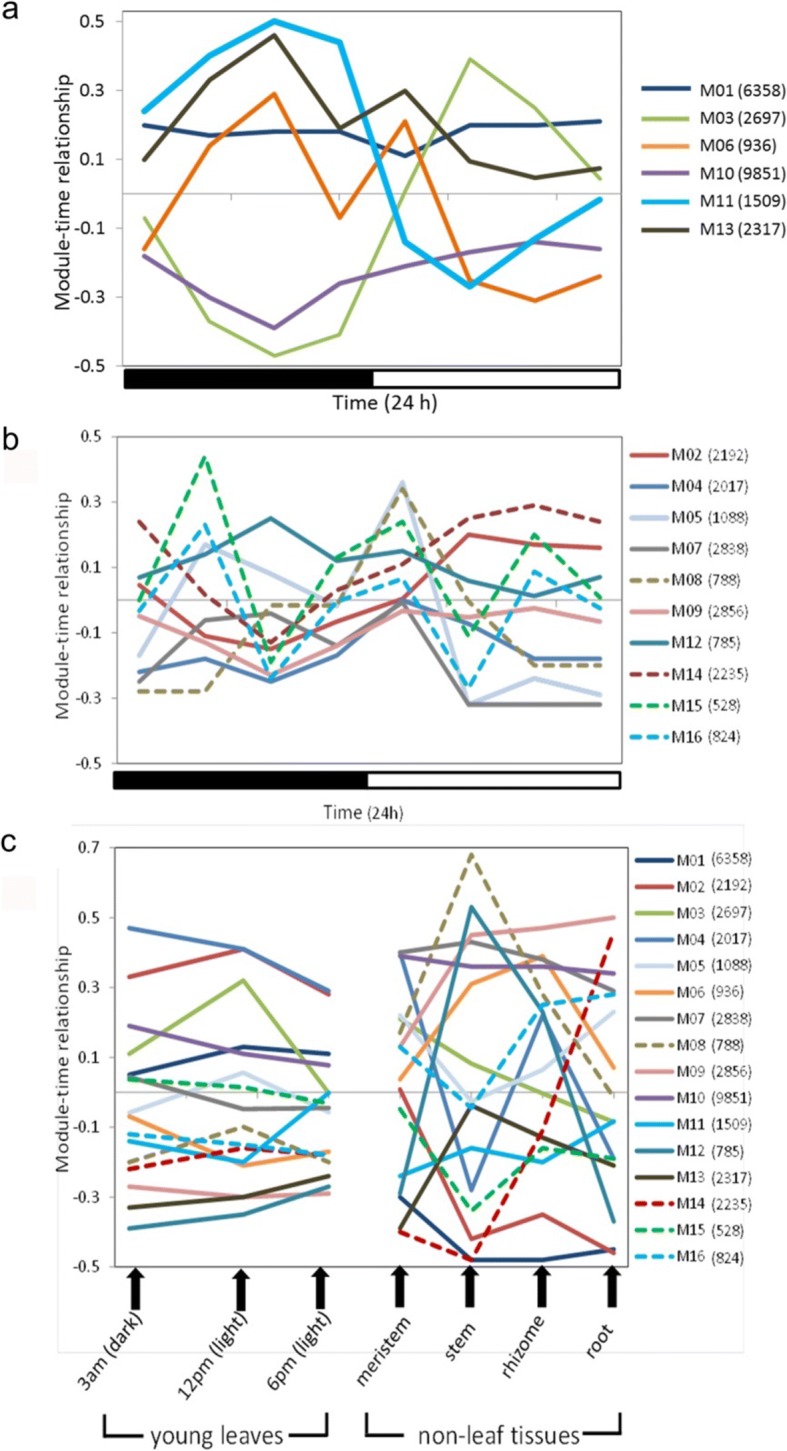
Temporal expression of CAM and gene co-expression modules in *Agave americana*. **a** Diel expression pattern of selected co-expression modules in mature leaf, as identified from network analysis of RNA-Seq data with relevance to CAM physiology. The black and white bars indicate nighttime and daytime, respectively. **b** Diel expression pattern of other modules in mature leaf with distinct profiles. **c** Expression pattern in young leaves sampled at 3 time points and non-leaf tissues sampled at one time point (9 am). The number in the parentheses is the number of transcripts in each individual module

Using the quantitative gene expression data obtained from the RNA-Seq analysis of 15 tissues, including mature leaf (sampled at 8 time-points over a diel cycle), young leaf (3 time-points), root, meristem, rhizome, and stem in *A. americana* cultivar ‘Marginata’ [14], we created a gene co-expression network that was partitioned into 16 co-expression modules, with each displaying distinctive diel patterns (Fig. 1). Gene ontology (GO) enrichment analysis identified biological processes over-represented (*p* < 0.05) in each of these co-expression modules (Additional file 1: Figure S1). We characterized the GO enrichment results together with expression profiles to identify potential modules for CAM. For example, module ‘M11’, containing 1509 transcripts, was significantly (*p* < 0.05) associated with CAM-defining nocturnal net CO_2_ uptake (9 pm – 6 am) (Fig. 1a), and was over-represented by biological processes relevant to stomatal movement, carboxylation, and signal transduction (Table 1). Module M11 contains some key genes involved in CAM metabolism, including *PHOSPHOENOLPYRUVATE CARBOXYLASE KINASE 1* (*PPCK1*), which regulates the temporal activation of nocturnal CO_2_ uptake. Since genes within module M11 are relevant to nocturnal carboxylation and stomatal movement, we propose that this module provides a molecular signature for temporal reprogramming of metabolism underpinning CAM.

**Table 1:** Biological processes over-represented (*p* < 0.02) in the co-expression module M11

GO ID	GO Term	Corrected *P*-Value
GO:0009738	Abscisic acid-activated signaling pathway	4.2E-08
GO:0016311	Dephosphorylation	6.2E-08
GO:0071215	Cellular response to abscisic acid stimulus	9.0E-08
GO:0009737	Response to abscisic acid	9.3E-08
GO:0097306	Cellular response to alcohol	5.1E-07
GO:0071396	Cellular response to lipid	6.6E-07
GO:0006470	Protein dephosphorylation	7.0E-07
GO:0097305	Response to alcohol	7.8E-07
GO:0009611	Response to wounding	4.6E-06
GO:0009745	Sucrose mediated signaling	4.8E-04
GO:0009753	Response to jasmonic acid	6.3E-04
GO:0019722	Calcium-mediated signaling	1.7E-03
GO:0009788	Negative regulation of abscisic acid-activated signaling pathway	1.8E-03
GO:1901420	Negative regulation of response to alcohol	1.8E-03
GO:0009694	Jasmonic acid metabolic process	2.5E-03
GO:1901419	Regulation of response to alcohol	2.6E-03
GO:0009787	Regulation of abscisic acid-activated signaling pathway	2.6E-03
GO:0009968	Negative regulation of signal transduction	2.7E-03
GO:0010648	Negative regulation of cell communication	2.7E-03
GO:0023057	Negative regulation of signaling	2.7E-03
GO:0019856	Pyrimidine nucleobase biosynthetic process	2.7E-03
GO:0010224	Response to UV-B	5.0E-03
GO:0019932	Second-messenger-mediated signaling	6.0E-03
GO:0010243	Response to organonitrogen compound	6.1E-03
GO:0006835	Dicarboxylic acid transport	6.3E-03
GO:0009875	Pollen-pistil interaction	6.3E-03
GO:0010200	Response to chitin	6.5E-03
GO:0009695	Jasmonic acid biosynthetic process	7.0E-03
GO:0006206	Pyrimidine nucleobase metabolic process	7.4E-03
GO:0006984	ER-nucleus signaling pathway	8.1E-03
GO:0071324	Cellular response to disaccharide stimulus	8.3E-03
GO:0071329	Cellular response to sucrose stimulus	8.3E-03
GO:0042538	Hyperosmotic salinity response	9.6E-03
GO:0030968	Endoplasmic reticulum unfolded protein response	1.1E-02
GO:0015743	Malate transport	1.2E-02
GO:0034620	Cellular response to unfolded protein	1.3E-02
GO:0035967	Cellular response to topologically incorrect protein	1.3E-02
GO:0010118	Stomatal movement	1.3E-02
GO:0006986	Response to unfolded protein	1.3E-02
GO:0048544	Recognition of pollen	1.5E-02
GO:0008037	Cell recognition	1.6E-02
GO:0009827	Plant-type cell wall modification	1.8E-02
GO:0015740	C_4_-dicarboxylate transport	1.9E-02

### Orthologous gene groups among CAM, C_3_ and C_4_ species

**Fig. 2:**
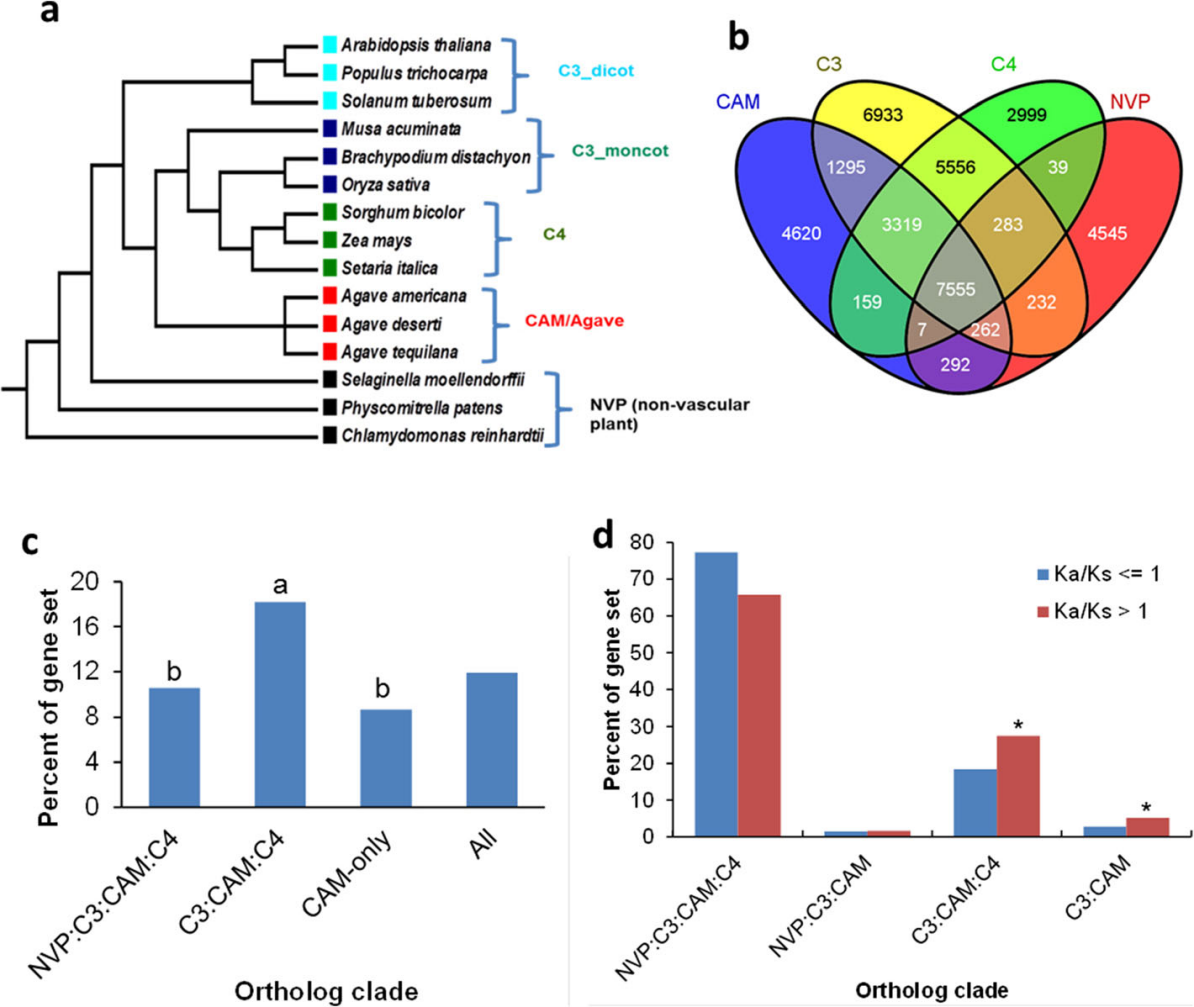
Comparative analysis of protein sequences among CAM and non-CAM plant species. **a** Plant species used in comparative genomics analysis. **b** Ortholog groups in 15 plant species as identified by OrthoMCL. Number of ortholog groups is listed in each of the ortholog clades. **c** Percent of ortholog clade were predicted to be transcription factors in *Agave americana*. “a” and “b” indicate that the transcription factors are over-represented (*p* < 0.05) and under-represented (*p* < 0.05), respectively. **d** Percent of ortholog clade undergoing positive selection (i.e., nonsynonymous to synonymous substitution ratio (Ka/Ks) > 1, as calculated from *Agave*-*Arabidopsis* gene pairs with a sliding window of 50 amino acids). “*” indicates that the ortholog clade was over-represented (*p* < 0.0001) by *Agave* genes with Ka/Ks ratio greater than 1. Clade NVP:C_3_:CAM:C_4_ is shared by NVP, C_3_, CAM, and C_4_; NVP:C_3_:CAM shared only by NVP, C_3_, and CAM; C_3_:CAM:C_4_ shared only by C_3_, CAM, and C_4_; C_3_:CAM shared only by C_3_ and CAM; and CAM-only is specific to CAM species

To understand the evolutionary origins and possible shared trajectories of module M11 and other CAM components between different photosynthetic lineages, we performed comparative genomics analysis of 15 plant species, including CAM, C_3_, C_4_, and non-vascular plant (NVP) species (Fig. 2a). Specifically, we identified ortholog clades through OrthoMCL analysis, such as clade NVP:C_3_:CAM:C_4_, which contains ortholog groups shared by NVP, C_3_, CAM, and C_4_ species, and clade C_3_:CAM:C_4_, which contains ortholog groups shared only by C_3_, CAM, and C_4_ species (Fig. 2b). The genes in the *Agave* (CAM) species were distributed mainly in three ortholog clades: NVP:C_3_:CAM:C_4_, C_3_:CAM:C_4_, and CAM-only, whereas the genes found in C_4_ species were distributed mainly in four ortholog clades: NVP:C_3_:CAM:C_4_, C_3_:CAM:C_4_, C_3_:C_4_, and C_4_-only (Additional file 2: Table S1), indicating that C_4_ evolution has one additional major genomic event, as represented by clade C_3_:C_4_, relative to CAM evolution.

Gene ontology enrichment analyses revealed that *A. americana* genes in clade NVP:C_3_:CAM:C_4_ were over-represented by biological processes relevant to primary metabolic processes (Additional file 3: Table S2), while those in clade C_3_:CAM:C_4_ were over-represented by regulatory processes (Additional file 4: Table S3). Notably, the core enzymes in C_4_ and CAM pathways belong to clade NVP:C_3_:CAM:C_4_, whereas the majority of the regulatory proteins belong to clade C_3_:CAM:C_4_ (Additional file 5: Table S4). Furthermore, genes in the M11 module were over-represented in the ortholog clade C_3_:CAM:C_4_ (Additional file 6: Table S5). Transcription factors were also over-represented (*p* < 0.05) in clade C_3_:CAM:C_4_, but under-represented (p < 0.05) in both NVP:C_3_:CAM:C_4_, and CAM-only clades (Fig. 2c). These results indicate that CAM evolution in *Agave* required genes that are shared across C_3_, C_4_ and CAM lineages to act as regulatory agents, whereas the core metabolic CAM machinery predates the C_3_-CAM-C_4_ divergence and is shared by NVP, C_3_, CAM, and C_4_ lineages.

Since the three CAM species (*A. americana*, *A. deserti* and *A. tequilana*) in this research are closely-related, the CAM-only ortholog groups may contains two types of CAM-specific genes: 1) specific to the *Agave* lineage and 2) conserved CAM-specific genes shared between *Agave* and other CAM lineages. To identify the conserved CAM-specific genes shared between *Agave* and other CAM lineages, the *Agave* genes in the CAM-only ortholog groups were compared with the protein tribes from the same 15 plant species as those used for ortholog group analysis, which were constructed by using TribeMCL [19]. In general, the protein tribes are equivalent to gene families, with each tribe containing multiple ortholog groups. The *A. americana* genes in both the CAM-only ortholog groups and CAM-only tribes were then compared with an extended list of CAM and non-CAM species using BLASTp, resulting in the identification of 13 *A. americana* genes that have homologs in other three independent CAM lineages (i.e., *Kalanchoë fedtschenkoi*, *Ananas comosus*, and *Phalaenopsis equestris*) but not in 21 non-CAM species (Additional file 7: Table S6). Some of these CAM-specific genes displayed variable diel expression patterns (Additional file 8: Figure S2).

### Positive selection in CAM evolution

**Fig. 3:**
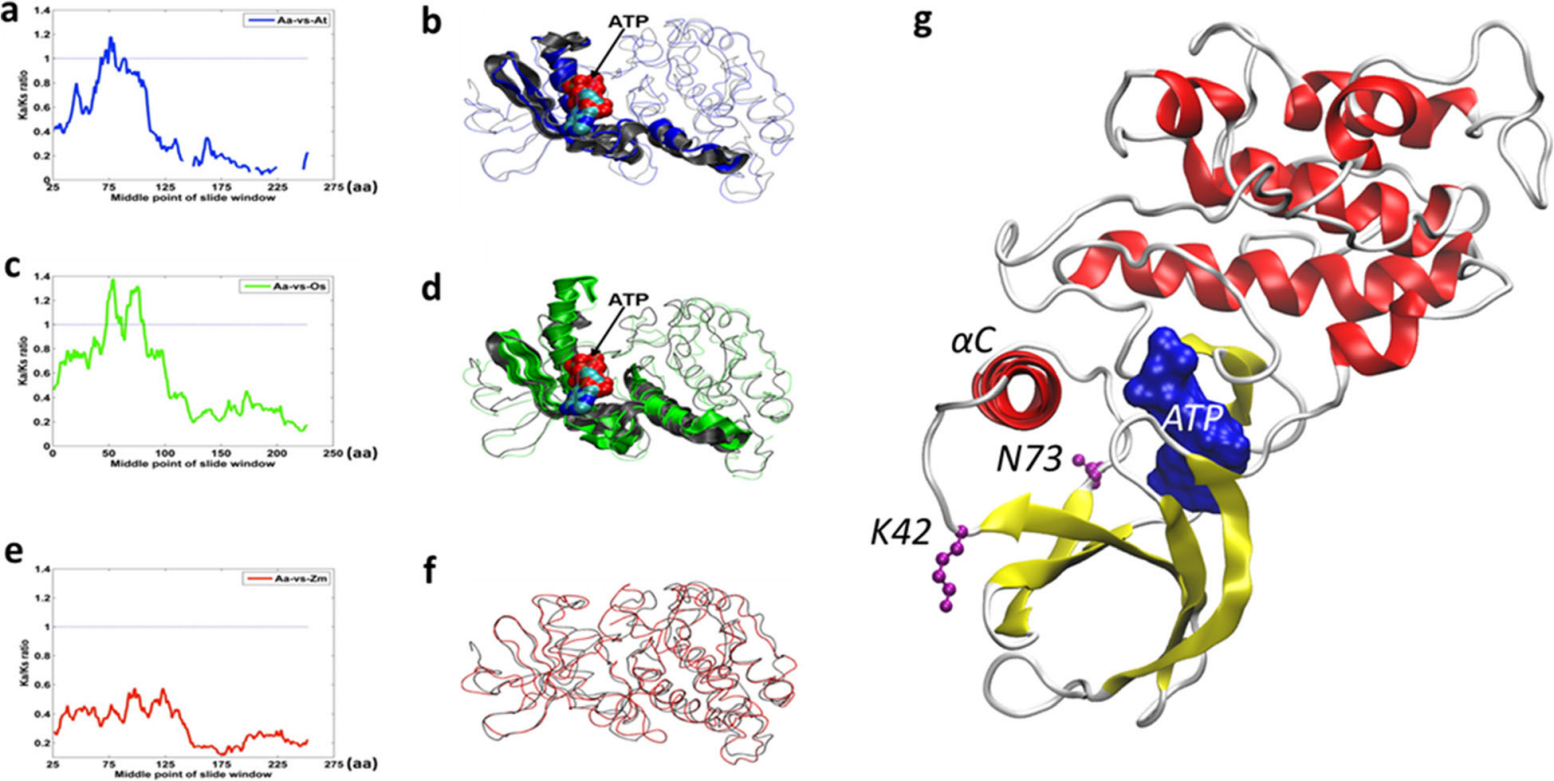
Positive selection region in phosphoenolpyruvate carboxylase kinase (PPCK1). **a** Ka/Ks profile of *Agave americana* (Aa) versus *Arabidopsis thaliana* (At); **b** superimposed structures in Aa and At, with the positive selection region highlighted; (**c**) Ka/Ks profile of Aa versus *Oryza sativa* (Os); **d** superimposed structures in Aa and Os, with the positive selection region highlighted; **e** Ka/Ks profile of Aa versus *Zea mays* (Zm); **f** superimposed structures in Aa and Zm. An ATP substrate that may bind to the Aa PPCK1 is marked by an arrow. The proteins are colored in grey for Aa, blue for At, green for Os and red for Zm. **g** A snapshot of PPCK1 (Aam048341) structure model revealing the positive selected sites. The PPCK1 model bound with an ATP substrate (blue surface) is after a 1-us MD simulation. K42 (codon 124, at the C-end of β3) and N73 (codon219, at the N-end of β4) are located at the two strands connecting to the αC helix, the orientation of which is known to be involved in the activation of the kinase

To study protein sequences evolution, we analyzed the non-synonymous to synonymous substitution ratio (Ka/Ks) of orthologous gene pairs between *A. americana* and three non-CAM species, including two C_3_ species (*Arabidopsis thaliana* and *Oryza sativa*) and one C_4_ species (*Zea mays*). A Ka/Ks ratio greater than 1 indicates positive selection or an acceleration of protein evolution [20, 21]. Ka/Ks analysis identified a set of 160 *Agave* genes that had protein sequence regions with Ka/Ks ratio greater than one, as compared with orthologous genes in the three non-CAM species (Additional file 9: Table S7), indicating that these genes experienced accelerated amino acid substitutions during the divergence between CAM and non-CAM species. The functionally annotated genes in this set included genes involved in circadian clock, starch and sugar metabolism, and decarboxylation (Additional file 10: Table S8). Importantly, we identified 94 *Agave* genes that had protein sequence regions with Ka/Ks ratio greater than 1, as compared with orthologous genes in the two C_3_ species, but not the orthologs in the C_4_ plant (Additional file 11: Table S9). The functionally annotated genes in this 94-gene set include *PPCK1* (Fig. 3a, c, and e), a core regulator of nocturnal carboxylation, and *PsI-D2* (Additional file 11: Table S9), which encodes a component of photosystem I essential for photosynthesis [22] and a chloroplast beta-amylase (*CT-BMY*).

To further consolidate the results of positive selection analysis based on Ka/Ks ratio, codon-based site analysis was performed to identify specific amino acid sites under positive selection. Out of the 94 genes that were revealed to experience positive selection by Ka/Ks ratio analysis (Additional file 11: Table S9), 64 genes were shown to carry at least one positively selected site, with posterior probabilities > 80% (Additional file 12: Table S10). We found 2 sites in PPCK1 (K42, N73) and 1 site in CT-BMY (E24) were supported by positive selection analysis based on both random effects likelihood (REL) and Fast, Unconstrained Bayesian AppRoximation (FUBAR) models (Table 2). Our 3-D protein structural modeling revealed that the positive-selection regions (sites) occur in important functional domains. The regions with Ka/Ks ratio > 1 of PPCK1 were located in the N-terminal domains responsible for ATP-binding (Fig. 3b and d), and K42 and N73 were likely involved in the activation of kinase (Fig. 3g). The positively selected regions of CT-BMY are responsible for substrate binding (Additional file 13: Figure S3). The clade C_3_:CAM:C_4_ was over-represented (*p* < 0.0001) by *Agave* genes with Ka/Ks ratio > 1 in the *Agave*-*Arabidopsis* gene pair comparison (Fig. 2d). Together, these results indicate that accelerated amino acid substitution has played a key role in the modification of proteins required for the light and carbon processing reactions of photosynthesis, as well as regulatory and signaling pathways in *Agave*.

**Table 2:** The positively selected sites of PPCK1 and CT-BMY under models from HYPHY (REL, FUBAR). The sites were listed as positively selected sites if they had a posterior probability greater than 80%

	Codon(FUBAR)	α	β	β-α	Posterior Prob β > α	Emp. Bayes Factor	PSRF	Neff
PPCK1	124*	0.81	2.82	2.01	0.83	10.81	1.00	1292.65
	219*	0.81	2.71	1.90	0.82	10.27	1.00	1327.29
CT-BMY	73*	0.60	1.93	1.34	0.83	15.97	1.01	491.79
	Codon(REL)	E[dS]	E[dN]	Normalized E[dN-dS]	Posterior Probability	Bayes Factor		
PPCK1	124*	0.96	3.43	2.47	0.95	307.87		
	219*	0.97	3.41	2.44	0.94	268.09		
	267	1.07	3.31	2.24	0.90	155.82		
	273	1.06	3.32	2.25	0.90	165.39		
	275	1.09	3.14	2.05	0.85	97.75		
	276	1.07	2.85	1.78	0.77	58.35		
CT-BMY	73*	0.94	0.85	−0.0961	0.75	52.6		

The * sites indicated they were identified by both models

### Diel re-programming of gene expression between CAM and C_3_

**Fig. 4:**
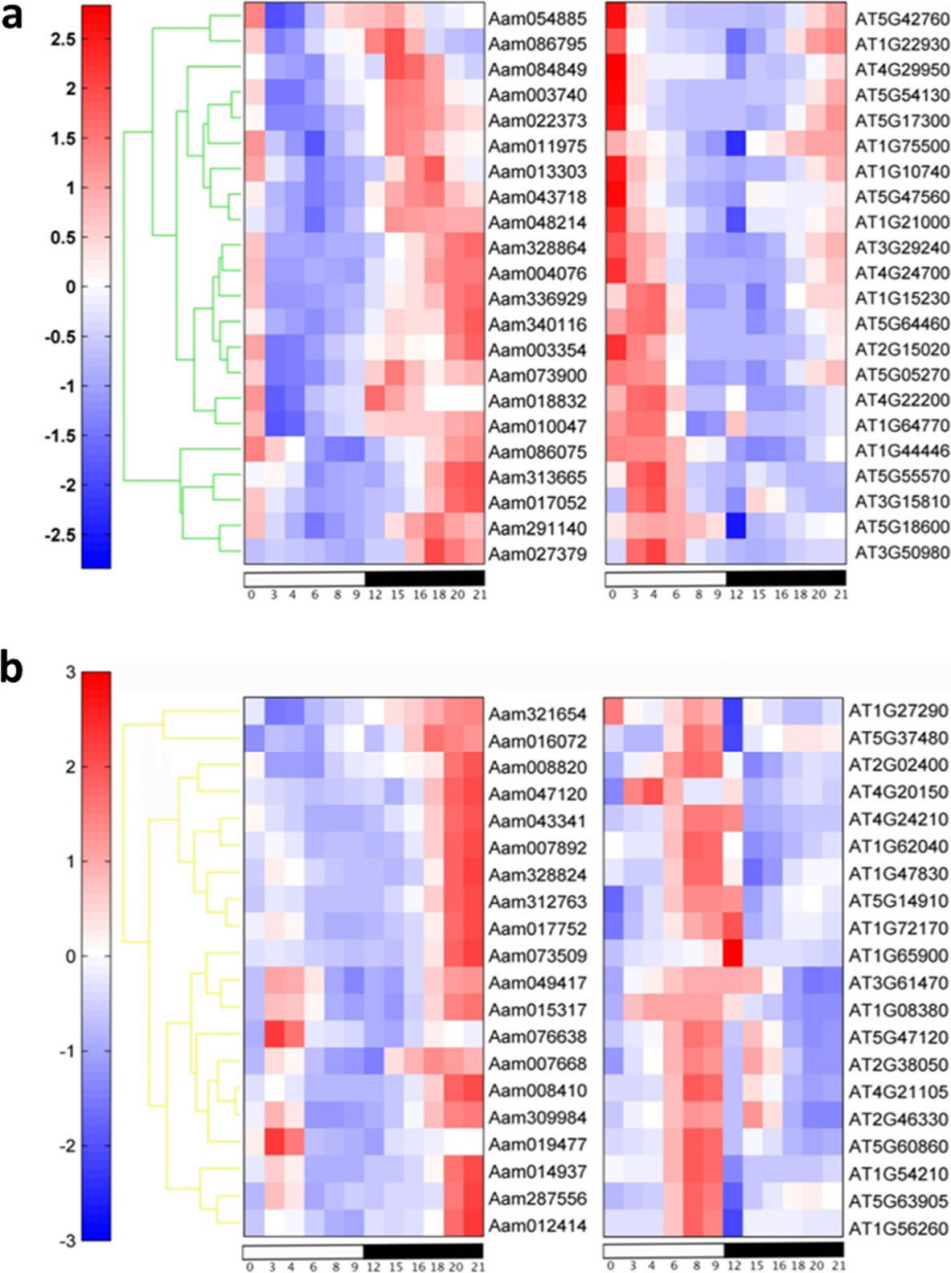
Diel shift in gene expression pattern between *Agave americana* and *Arabidopsis thaliana*. **a** Morning-to-night shift with peak expression during morning in *Arabidopsis* and during night in *Agave*. **b** Afternoon-to-night shift with peak expression during afternoon in *Arabidopsis* and during late night in *Agave*. See gene annotation in Additional file 14: Table S11 and Additional file 15: Table S12

To further examine the molecular basis of the diel re-programming of metabolism that underpins CAM, we performed a comparative analysis of time-course expression data between *A. americana* (CAM) and *Arabidopsis thaliana* (C_3_). We identified two clusters of *Agave* genes that exhibited shifts in day/night patterns of abundance relative to the corresponding orthologous genes in *Arabidopsis*. One cluster, containing 22 genes, showed a morning-to-night shift with alternative peak expression at night and morning between *Agave* and *Arabidopsis*, respectively (Fig. 4a). This gene set was over-represented (*p* < 0.05) by co-expression modules M01 and M11 (Table 3), with M11as the aforementioned molecular marker for CAM-associated nocturnal gene expression. Among the 22 genes showing the morning-to-night shift, 8 encode proteins with unknown function; the 14 annotated genes have functions related to circadian clock, photosynthetic electron transport, malate transport, stomatal movement, and redox homeostasis (Additional file 14: Table S11). The other cluster, containing 20 genes, showed an afternoon-to-night shift, with alternate peak expression during late night and afternoon in *Agave* and *Arabidopsis*, respectively (Fig. 4b). This gene set was over-represented by co-expression modules M06 (*p* < 0.05) and M13 (*p* < 0.01) (Table 3), both of which showed positive association with gene expression in mature leaves around midnight (Fig. 1b), suggesting that the genes in these two modules are involved in CAM related processes during the night. Among the 20 genes showing the afternoon-to-night shift, 7 encode proteins with unknown function; 13 have known functions related to signaling, sugar metabolism and light processing (Additional file 15: Table S12). In concern of multiple copies of *Agave* and *Arabidopsis* genes in an ortholog group, we assessed each ortholog group containing abovementioned genes in Fig. 4, and found 23 ortholog groups containing two genes, with a one-to-one relationship between *Agave* and *Arabidopsis*. We evaluated the phylogeny and diel expression pattern for ortholog groups with a total of more than two *Agave* and *Arabidopsis* genes, and found that the expression patterns of *Agave* genes in comparison with their orthologs in *Arabidopsis* displayed differential day-night patterns (Additional file 16: Figure S4 and Additional file 17: Figure S5), suggesting a functional diversification of gene family members between C_3_ and CAM plants.

**Table 3:** Distribution of gene co-expression modules in the gene sets with positive selection and diel shift in gene expression pattern, respectively, in *Agave americana*. Gene set 1a includes *Agave* genes with Ka/Ks ratio greater than one in the *Agave*-*Arabidopsis*, *Agave*-*Oryza* and *Agave*-*Zea* orthologous gene pairs. Gene set 1b includes *Agave* genes with Ka/Ks ratio greater than one in *Agave*-*Arabidopsis* and *Agave*-*Oryza* but not *Agave*-*Zea* pairs. Gene sets 2a and 2b includes *Agave* genes with morning-to-night shift and afternoon-to-night shift, respectively, in expression pattern as compared with the orthologous genes in *Arabidopsis*. The numbers represent the observed and expected (in parentheses) number of genes

Co-expression module	Gene set 1a	Gene set 1b	Gene set 2a	Gene set 2b
M01	14 (27)	17 (16)	10 (4)*	5 (4)
M02	13 (7)*	4 (4)	2 (1)	0 (1)
M03	16 (7) **	10 (4)*	0 (1)	0 (1)
M04	1 (5)	0 (3)	0 (1)	0 (1)
M05	0 (4)	0 (2)	1 (1)	0 (1)
M06	1 (4)	1 (2)	0 (1)	3 (1)*
M07	5 (10)	1 (6)	1 (1)	2 (1)
M08	2 (2)	0 (1)	0 (0)	0 (0)
M09	1 (9)	2 (5)	0 (1)	1 (1)
M10	49 (28)**	23 (16)	0 (4)	0 (4)
M11	5 (5)	2 (3)	4 (1)*	0 (1)
M12	0 (3)	0 (2)	0 (0)	0 (0)
M13	3 (11)	2 (6)	3 (2)	10 (1)**
M14	2 (8)	2 (5)	0 (1)	0 (1)
M15	0 (2)	1 (1)	0 (0)	0 (0)
M16	1 (2)	1 (1)	1 (0)	0 (0)
Non-module	47 (25)**	28 (15)*	0 (4)	0 (3)
Total	160	94	22	21

^*^Overrepresentation (FDR adjusted *p*-value< 0.05, cumulative Poisson distribution) of co-expression modules in each category. ^**^Overrepresentation (FDR adjusted *p*-value< 0.01, cumulative Poisson distribution) of co-expression modules in each category

### Comparison of circadian clock pathway between CAM and C_3_

**Fig. 5:**
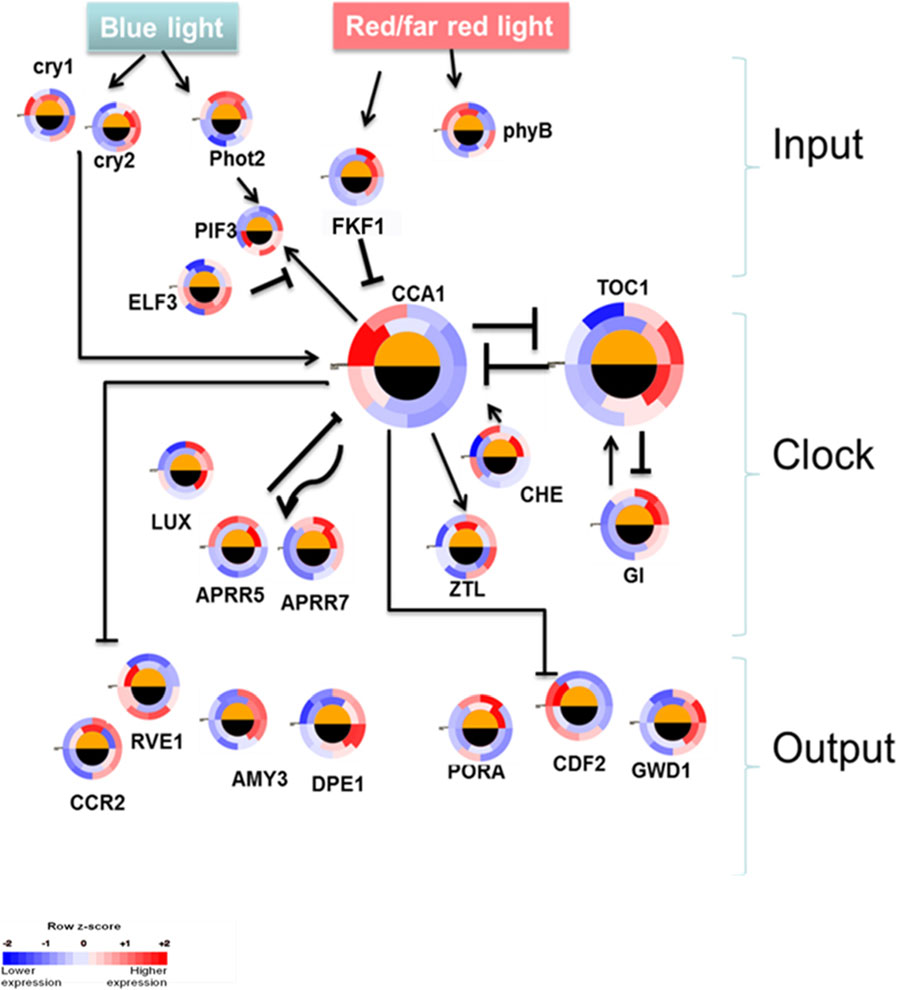
Diel gene expression pattern of circadian system genes in *Agave americana* and *Arabidopsis thaliana*. In the circular heatmaps, the outer and inner rings represent *Agave americana* and *Arabidopsis thaliana*, respectively. The black and white half-circles inside the circular heatmaps indicate night-time and day-time, respectively. Full gene names are listed in Additional file 18: Table S13

**Fig. 6:**
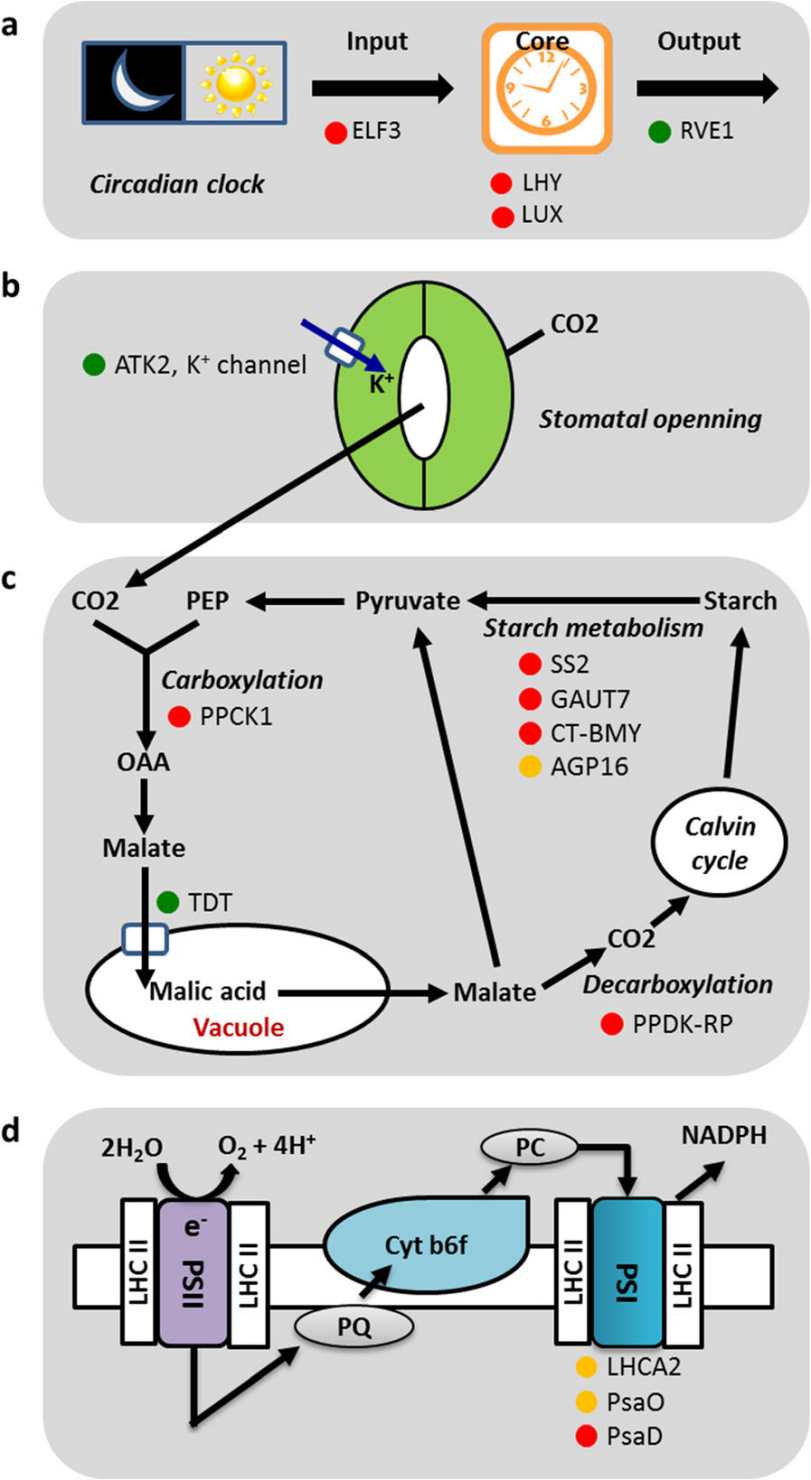
Functionally annotated *Agave* genes showing positive selection and diel rewiring of expression pattern relative to C_3_ plants. **a** Genes involved in circadian clock. A gene involved in stomatal opening. **c** Genes involved in carboxylation, malate transport, decarboxylation, and starch/sugar metabolism. **d** Genes involved in photosynthetic electron transport chain. Red circles indicate positive selection. Green circles indicate morning-to-night shift in peak gene expression. Yellow circles indicate afternoon-to-night shift in peak gene expression. AGP16, Arabinogalactan protein 16; AKT2, Arabidopsis Shaker family K^+^ channels 2/3; CT-BMY, Chloroplast Beta-Amylase; ELF3, Early Flowering 3; GAUT7, Galacturonosyltransferase 7; LHY, Late Elongated Hypocotyl; LUX, Phytoclock 1; PPCK1, Phosphoenlpyruvate Carboxylase Kinase 1; PPDK-RP, Pyruvate orthophosphate dikinase regulatory protein; SS2, Starch Synthase 2; TDT, Tonoplast Dicarboxylate Transporter

We compared the diel expression pattern of genes implicated in signal input to the clock (e.g., *PHOT2*, *Phototropin-2*), clock oscillation (e.g., *CCA1*, *Circadian Clock Associated 1*; *TOC1*, *TIMING OF CAB EXPRESSION1*), and regulatory output from the clock (e.g., *RVE1*, *REVEILLE 1*) between *Agave* (CAM) and *Arabidopsis* (C_3_). All known genes implicated in input to the clock and the central clock oscillator showed similar diel expression patterns between *Agave* and *Arabidopsis*. However, *RVE1* exhibited patterns of peak transcript abundance that were substantially out of phase between CAM and C_3_, with the peak expression of *RVE1* occurring at midnight in *Agave* and morning in *Arabidopsis* (Fig. 5 and Fig. 6a; Additional file 18: Table S13). These comparative analyses support the concept that the multiple independent evolutionary origins of CAM exploited an existing C_3_ multi-gene loop oscillator similar to that in *Arabidopsis thaliana*, and diel re-programming of metabolism was achieved via changes to genes like *RVE1* that link metabolic output to the clock.

## Discussion

### Regulatory genes are critical for C_3_-to-CAM evolution

The distribution of CAM plants in diverse phylogenetic lineages indicates CAM has evolved from C_3_ via convergent evolution [23]. This wide-spread convergent evolution could lead to a hypothesis that C_3_-to-CAM evolution is relatively easy, not requiring whole-genome-scale changes. In support of this hypothesis, the results in this study indicate that CAM evolution required genes that are shared across C_3_, C_4_, and CAM lineages to act as regulatory agents, whereas the core metabolic CAM machinery predates the CAM-C_4_ divergence and is shared by NVP, C_3_, CAM, and C_4_ lineages. The data presented here have shown that genes encoding enzymes required for C_3_ and C_4_ carboxylation, decarboxylation, and carbohydrate processing, as well as membrane transporters required for intercellular trafficking of metabolites required for CAM, were present across all the plant lineages examined. Thus, comparative analysis of protein sequences revealed that the core metabolic CAM machinery predates the CAM-C_4_ divergence and is shared by non-vascular plants (NVP), C_3_, CAM, and C_4_ lineages. Such data is also consistent with recent reports that PEPC had shared origins in C_4_ and CAM lineages before the divergence of these two pathways from a C_3_ progenitor [24].

As a means of focusing in on the evolution of the regulatory components required for the diel re-programming of metabolism that defines CAM, gene co-expression network analysis was used to reveal modules with distinctive diel patterns of abundance. One gene module in particular, designated M11, was identified as providing a molecular signature for the temporal re-programming of metabolism underpinning CAM. Gene module M11, showed abundant expression in mature leaves at night and contained *PPCK1*, which regulates the temporal activation of nocturnal CO_2_ uptake by PEPC [25, 26]. Module M11 was also over-represented by biological processes relevant to stomatal movement and signal transduction. In particular, gene ontology terms related to the ABA signaling pathway were significantly enriched in M11 (Table 1). Such genes are commonly known for their key roles in stomatal regulation and responses to stress [27]. It is also noteworthy that sucrose and jasmonic acid signaling pathways were also enriched in M11, suggesting the involvement of hormones and/or metabolites in the regulation of nocturnal CO_2_ uptake in CAM. The genes in M11, along with multiple transcription factors, were over-represented in the ortholog clades shared across C_3_, C_4_ and the *Agave*/CAM lineages. Therefore, it can be hypothesized that a limited number of key regulators can drive the C_3_-to-CAM transition. To test this hypothesis, future studies should focus on detailed functional characterization of the transcription factors in module M11, which belong to the ortholog clade shared across C_3_, C_4_, and CAM lineages. Such approaches will be critical for accelerating efforts designed to engineer CAM into C_3_ crops [13, 16].

### Regulation of stomatal movement is critical for CAM evolution

The altered night/day opening/closing of stomata is a core feature of CAM which requires coordination between mesophyll and guard cells [28]. Genes related to stomatal movement were found to be enriched in module M11, alongside genes relevant for nocturnal carboxylation (Table 1). Guard cell inward-rectifying K^+^ channel AKT2 plays an important role in light-induced stomatal opening in *Arabidopsis* [29]. Our analysis revealed that the peak expression of *AKT2* was shifted to the night in *Agave* (CAM), as compared with day-time peak expression in *Arabidopsis* (C_3_) (Fig. 4a; Fig. 6b), suggesting that AKT2 is involved in nocturnal stomatal opening in *Agave*. In terms of determining the signals that might be responsible for shifting the timing of AKT2 expression in CAM, it has been suggested that photosynthetic metabolism in the mesophyll cells could contribute to the regulation of guard cell function [30]. Interactions between mesophyll photosynthesis and guard cell regulation have been revealed in many non-CAM species [31]. In particular, signals driven by sugar and malate content in the mesophyll appear to have central roles in controlling stomatal aperture [31]. In C_3_ plants, malate metabolism in the mesophyll and malate transport from mesophyll to guard cells has been shown to play a central role in regulating stomatal responses over the day/night cycle [16]. By analogy, it can be hypothesized that in CAM plants, the diel turnover and transport of malate across the vacuolar tonoplast membrane will play a critical role in stomatal regulation [16]. In *Arabidopsis*, tonoplast dicarboxylate transporter (TDT) imports malate into the vacuoles [32]. In *Agave*, the peak expression of *TDT* was shifted to the night, as compared to peak expression during the day in *Arabidopsis* (Fig. 4a; Fig. 6c), implying that the *Agave* TDT is responsible for the transport of malate into the vacuole during the dark period. Future molecular genetic experiments are needed to confirm the function of *AKT2* and *TDT*, and consequently shed new light on identifying signals which integrate carboxylation processes with stomatal movement in CAM.

### Diversification of circadian clock genes in CAM

Our results suggest that circadian shifts in gene transcription underpin the CAM-defining diel patterns of stomatal conductance, malate transport, carbohydrate processing and supply, and demand for ATP and reducing power in *Agave*. The circadian clock has been proposed to control the extensive re-synchronization of metabolism that distinguishes CAM from C_3_ and C_4_ photosynthesis [4]. Circadian rhythms are ubiquitous in eukaryotes and many features of the circadian clock are conserved across plant lineages [33]. Genes in the circadian system have been well-studied in *Arabidopsis* and can be divided into three functional groups: signal input (e.g., *PHOT2*), clock oscillation (e.g., *CCA1*, *TOC1*), and regulatory output (e.g., *RVE1*) [34]. We identified homologs in *Agave* (CAM plant) for all the known clock genes in *Arabidopsis* (C_3_) and found that the diel expression patterns of all the clock genes are conserved between CAM and C_3_, except for *RVE1* (a Myb-like transcription factor) in the output subset (Fig. 5), suggesting that C_3_ and CAM plants share the same core circadian oscillator, with diversification occurring in the regulatory output from the core clock, such as *RVE1*. It was previously reported that *RVE1* integrates the circadian clock and auxin pathways to coordinate plant growth with changes in environmental time cues in *Arabidopsis* [35]. Here we hypothesize that *RVE1* is a key a node from which the C_3_ and CAM clock diverge and rewiring the diel expression of *RVE1* is one of the necessary steps to switch from C_3_ to CAM.

Besides expression analysis, changes in the protein sequences of circadian clock components may also contribute to the diversification of clock functions. We performed 3D structural modeling to understand the function of protein domains containing sites of positive selection (reflected by Ka/Ks ratio > 1). Our structure-modeling revealed characteristics of positive-selection in the intrinsically disordered protein regions in some circadian clock proteins (Additional file 19: Figure S6). It has become clear that a certain protein may not have a well-defined and compactly folded three-dimensional (3D) structure under physiological conditions and such proteins are often termed natively unfolded protein [36] or an intrinsically disordered protein (IDP) [37]. Many intrinsically disordered protein regions (IDPRs) have been known to carry out important biological functions [38]. The clock proteins Late Elongated Hypocotyl (LHY), Early Flowering 3 (ELF3), and Lux Arrhythmo (LUX) play key roles in the plant circadian oscillation [39]. The Ka/Ks profile of each of these circadian rhythm proteins showed multiple positive selection regions with significant fluctuations in *Agave* (data not shown). Interestingly, these proteins were found to exhibit a high ratio of IDPRs with high PONDR scores (Additional file 20: Table S14). The IDPRs of the clock proteins may lead to high flexibility in both structure and function, which in turn, could favor novel interactions with nucleic acids and/or other proteins. Further molecular genetics studies are needed to gain deeper understanding of the functions encoded in the apparent disordered state of these clock proteins and their role in the evolution of CAM.

### CAM-specific genes and evolution

Multiple lineages of CAM photosynthesis plants have evolved independently from C_3_ photosynthesis ancestors [13, 40]. Recently, a comparative study using four genera in subfamily Agavoideae with CAM, weak CAM and C_3_, has suggested that gene family analysis together with expression profiling is informative in understanding the divergence of CAM [41]. However, a broader sampling of diverse CAM lineages is still necessary. It can be hypothesized that there are two types of CAM-specific genes: 1) lineage-specific CAM genes shared by multiple closely related-species (e.g., *Agave* spp.) and 2) conserved CAM-specific genes shared by multiple independent lineages of CAM plants. To test this hypothesis, it would be useful in the future to expand this work by including several independently evolved CAM lineages in order to separate evolutionary phenomena unique to the CAM *Agave* from ones shared by different CAM lineages.

## Conclusions

Evidence is presented that the genetic components of core CAM machinery in *Agave* have an ancient origin traceable to non-vascular plant lineages, and that regulatory proteins, which are shared between C_3_, CAM, and C_4_ species, were essential to the C_3_-to-CAM transition. The evolution of CAM in *Agave* from C_3_ photosynthesis also required positive selection in protein sequences of enzymes and transporters implicated in metabolism and signaling associated with CAM, as well as diel re-programming of gene expression related to key biological processes, such as circadian rhythms, redox homeostasis, and carbohydrate metabolism (Fig. 6). These results provide a set of new candidate genes for engineering increased water-use efficiency in crop plants experiencing water-limiting conditions via synthetic biology approaches.

## Methods

### Representative protein model per locus in *Agave* species

The transcript sequences of *A. americana* [14] (Additional file 21) were first filtered by CD-HIT-EST [42, 43] with a sequence identity threshold of 0.98 and the alignment coverage for the shorter sequence set as 0.5. This was an optimal setting based on the test with various combinations of sequence identity (0.90, 0.91, …, 1.0), and the alignment coverage for the shorter sequence (0.4, 0.5, …, 0.8), using the *Arabidopsis* genome annotation (TAIR10). To assign the representative protein model per locus with high-confidence, the protein sequences corresponding to the representative transcript sequences in *A. americana*, obtained from CD-HIT-EST clustering, as well as the non-redundant representative protein models of *A. deserti* and *A. tequilana* [44], were mapped onto the aforementioned draft genome assembly of *A. tequilana* using BLAT [45] with a minimum coverage (i.e., minimum fraction of query that must be aligned) of 60% and a minimum identity of 90%. Only the “best match” position was selected as the genomic location for each query protein sequence. If multiple proteins mapped to the overlapping genome locations and they shared significant sequence similarity, as determined by BLASTp [46, 47] with E-value cutoff of 1e-5, the longest protein sequence was selected as the representative protein model for the gene locus, resulting in 55,451, 31,761, and 31,799 representative protein sequences in *A. americana*, *A. deserti* and *A. tequilana*, respectively (Additional files 22, 23 and 24).

### Comparative analysis of protein sequences

The protein sequences of 15 plant species, including the aforementioned representative protein sequences in three CAM species of *Agave* (*Agave americana, A. deserti* and *A. tequilana*), and 12 non-CAM plant species downloaded from public databases, which included three non-vascular plant species *Chlamydomonas reinhardtii* (www.Phytozome.net; Phytozome v9.0), *Physcomitrella patens* (Phytozome v9.0), *Selaginella moellendorffii* (Phytozome v9.0); three C_4_ plant species *Sorghum bicolor* (Phytozome v9.0)*, Setaria italica* (Phytozome v9.0) and *Zea mays* (Phytozome v9.0); three C_3_ monocot plant species *Brachypodium distachyon* (Phytozome v9.0e)*, Oryza sativa* (Phytozome v9.0), and *Musa acuminata* (version 1; http://banana-genome.cirad.fr); three C_3_ dicot species *Arabidopsis thaliana* (v10; www.*Arabidopsis*.org), *Populus trichocarpa* (Phytozome v9.0), and *Solanum tuberosum* (DM_v3.4; potatogenomics.plantbiology.msu.edu). The longest protein sequence was selected in case of multiple transcripts annotated for one gene locus. The ortholog groups (OGs) were constructed using OrthoMCL [48] with default parameters (a BLASTp E-value cutoff of 1e-5 and percent match cutoff of 50%). Also, the protein sequences used for ortholog analysis were clustered into tribes using TRIBE-MCL [19], with a BLASTp E-value cutoff of 1e-5 and an inflation value of 1.5. To identify conserved CAM-specific genes, the *A. americana* genes in both the CAM-only ortholog groups and CAM-only tribes were then compared with an extended list of three independent CAM lineages and 21 non-CAM species using BLASTp [46, 47] with an E-value cutoff of 1e-5. The three independent CAM lineages are *Kalanchoë fedtschenkoi* [49], *Ananas comosus* [50] and *Phalaenopsis equestris* [51]. The 21 non-CAM species are *Amborella trichopoda* (PLAZA 3.0 [52]; available at http://bioinformatics.psb.ugent.be/plaza/), *Arabidopsis thaliana* (PLAZA 3.0), *Beta vulgaris* (PLAZA 3.0), *Brachypodium distachyon* (PLAZA 3.0), *Carica papaya* (PLAZA 3.0), *Citrus sinensis* (PLAZA 3.0), *Eucalyptus grandis* (PLAZA 3.0), *Fragaria vesca* (PLAZA 3.0), *Medicago truncatula* (PLAZA 3.0), *Mimulus guttatus* (PLAZA 3.0), *Musa acuminata* (PLAZA 3.0), *Oryza sativa* (PLAZA 3.0), *Populus trichocarpa* (PLAZA 3.0), *Prunus persica* (PLAZA 3.0), *Setaria italica* (PLAZA 3.0), *Solanum lycopersicum* (PLAZA 3.0), *Solanum tuberosum* (PLAZA 3.0), *Sorghum bicolor* (PLAZA 3.0), *Theobroma cacao* (PLAZA 3.0), *Vitis vinifera* (PLAZA 3.0), *Zea may* (PLAZA 3.0).

### Co-expression network

A total of 47,677 transcripts that were detected in at least 4 of the 15 samples, with an average expression level of 5 RPKM or higher (Additional file 25), were utilized to construct a weighted gene co-expression network using the R package WGCNA [53]. The gene expression data were log2 transformed. The dynamic tree-cut algorithm was used to identify co-expression modules with a minimum module size of 30 and a height cut of 0.25.

### Comparative analysis of gene expression patterns between CAM and C_3_ plants

The *Arabidopsis*–*Agave* orthologous gene pairs were identified through the combination of both OrthoMCL strategies and the reciprocal best hits (RBH) based on BLASTp with an E-value cutoff of 1e-5. The diurnal expression data for *Arabidopsis thaliana* were obtained from Mockler et al. (2007) [9]. Both *Arabidopsis* and *Agave* plants were grown under a photoperiod of 12 h light:12 h dark cycle. The *Arabidopsis* expression data were collected at 0, 4, 8, 12, 16, 20, and 24 h, whereas the *Agave* data were collected at 0, 3, 6, 9, 12, 15, 18, and 21 h after the start of the light period [14]. The cubic interpolation algorithm implemented in Matlab (Mathworks, Inc.) was used to simulate the gene expression levels at additional time points, so that both time-course data sets consisted of the same time points: 0, 3, 4, 6, 8, 9, 12, 15, 16, 18, 20, and 21 h after the start of the light period. The gene expression data were normalized by Z score transformation. The hierarchical clustering of gene expression was performed using the Bioinformatics Toolbox in Matlab (Mathworks, Inc.).

### Gene ontology analysis

Whole-genome GO term annotation was performed using Blast2GO with a BLASTp E-value hit filter of 1 × 10–6, an annotation cutoff value of 55, and GO weight of 5. GO enrichment analysis for the ortholog clades was performed using BiNGO [54]. In addition, GO enrichment analysis was performed on each of the 16 co-expression modules using ClueGO [55] to interpret functionally grouped gene ontology annotation networks. The right-sided hypergeometric enrichment test was performed at a medium network specificity selection, and *p*-value correction was performed using the Benjamini-Hochberg method. The selected GO tree levels were a minimum of 3 and a maximum of 8, while each cluster was set to a minimum of between 3 and 4% genes. The GO term grouping setting was selected to minimize GO term redundancy, and the highest significance term enriched was used as the representative term for each functional cluster. The GO terms with *p*-values less than or equal to 0.05 were considered significantly enriched.

### Annotation of pathway and transcription factors

Pathway annotation for the protein sequences was performed on the KEGG Automatic Annotation Server KAAS [56], using the BBH (bi-directional best hit) method to assign orthologs. Transcription factors were identified from the protein sequences using the online tool PlantTFcat [57].

### Nonsynonymous (Ka) to synonymous (Ks) substitution ratio and positively selected sites

The orthologous gene pairs between two species were identified through the combination of both Best Reciprocal Hits (BRH) and OrthoMCL strategies. The coding sequences were aligned using PAL2NAL [58], guided by protein sequence alignment generated by MAFFT (linsi; version 7.045b) [59], and gaps in the alignment were removed. The gapless coding sequence alignments were used for Ka/Ks ratio calculation using the Bioinformatics Toolbox in Matlab (Mathworks, Inc.) with a 50-codon sliding window. For identifying positively selected sites, coding sequences from *Arabidopsis*, maize, rice and *Agave* were aligned by Translatorx [60] using the standalone script. The HyPhy package were used to identify positively selected sites as described [61], and the tests of FUBAR and REL models as implemented in Datamonkey webserver were used with default settings [62]. Since we used a sliding window to study the regions of protein with positive selection, we calculated the probabilities of Ka/Ks positive regions to a null hypothesis that Ka/Ks equals to one by one-sided t-test, as described by Schmid and Yang (2008) [63].

### Protein structure modeling

Protein structure models were built using the iterative threading assembly refinement (I-TASSER, version 3.0) methods [64]. The structure-based annotation tool COFACTOR [65] was adopted to predict the potential function and the cofactor binding site of the models. Disordered region(s) were analyzed using the PONDR VL-XT program [66–68].

### Phylogenetic tree construction

The multiple sequence alignment of protein sequences was created using MAFFT [59]. The phylogenetic tree was constructed from the protein sequence alignment using the Neighbor-Joining method [69] implemented in MEGA7 [70], with the percentage of replicate trees calculated by the bootstrap test (100 replicates). For trees with three sequences, no bootstrap value was given due to the lack of phylogeny tests of branch. All ambiguous positions were removed for each sequence pair.

## Additional files

Additional file 1: **Figure S1.** Overview charts of the gene ontology (GO) biological processes over-represented in individual co-expression modules. (PDF 464 kb)

Additional file 2: **Table S1.** Percentage of the gene set in each individual species distributed into different ortholog clades. (PDF 39 kb)

Additional file 3: **Table S2.** Biological processes over-represented (*p* < 1E-20) in ortholog clade NVP:C_3_:CAM:C_4_ in *Agave americana*. NVP:C_3_:CAM:C_4_ represents orthologs shared by C_3_, CAM, C_4_ and NVP (i.e. non-vascular plants). (PDF 39 kb)

Additional file 4: **Table S3.** Biological processes over-represented in ortholog clade C_3_:CAM:C_4_ in *Agave americana*. C_3_:CAM:C_4_ represents orthologs shared only by C_3_, CAM and C_4_ species. (PDF 17 kb)

Additional file 5: **Table S4.** Ortholog clades of C_4_ cycle genes & related transporters. (PDF 118 kb)

Additional file 6: **Table S5.** Distribution of co-expression modules in each individual ortholog clade in *Agave americana*. (PDF 90 kb)

Additional file 7: **Table S6.** List of *Agave americana* genes that have homologs in other three independent CAM lineages but not in 21 non-CAM plant species. (PDF 31 kb)

Additional file 8: **Figure S2.** The diel expression pattern of conserved CAM-specific genes in *Agave*. (PDF 111 kb)

Additional file 9: **Table S7**. List of *Agave americana* genes with Ka/Ks ratio greater than one in *Agave*-*Arabidopsis*, *Agave*-*Oryza* and *Agave*-*Zea* pairs. (PDF 353 kb)

Additional file 10: **Table S8.** Genes implicated in the CAM pathway undergoing positive selection as revealed from Ka/Ks ratio calculated from *Agave*-*Arabidopsis*, *Agave*-*Oryza* and *Agave*-*Zea* orthologous gene pairs. (PDF 91 kb)

Additional file 11: **Table S9.** List of *Agave americana* genes with Ka/Ks ratio greater than one in *Agave*-*Arabidopsis* and *Agave*-*Oryza* but not *Agave*-*Zea* pairs. (PDF 47 kb)

Additional file 12: **Table S10.** The positively selected sites of candidates with Ka/Ks > 1 from Table S9. (PDF 304 kb)

Additional file 13: **Figure S3.** Positive selection region in chloroplast β-amylase (CT-BMY). (PDF 171 kb)

Additional file 14: **Table S11.** List of *Agave americana* genes with morning-to-night shift in expression pattern as compared with the orthologous genes in *Arabidopsis*. (PDF 70 kb)

Additional file 15: **Table S12**. List of *Agave americana* genes with afternoon-to-night shift in expression pattern as compared with the orthologous genes in *Arabidopsis*. (PDF 69 kb)

Additional file 16: **Figure S4.** Phylogenetic trees and diel gene expression patterns of multi-gene ortholog groups (i.e., with a total of more than two *Agave* and *Arabidopsis* genes) listed Fig. 4a. (PDF 164 kb)

Additional file 17: **Figure S5.** Phylogenetic trees and diel gene expression patterns of multi-gene ortholog groups (i.e., with a total of more than two *Agave* and *Arabidopsis* genes) listed Fig. 4b. (PDF 156 kb)

Additional file 18: **Table S13.** List of circadian clock genes in *Agave americana* and *Arabidopsis thaliana*. (PDF 18 kb)

Additional file 19: **Figure S6.** Intrinsically disordered protein regions (IDPRs) and structural models of the circadian clock proteins in *Agave americana*. (PDF 153 kb)

Additional file 20: **Table S14.** Intrinsically disordered protein region (IDPR) in protein sequences under positive selection. (PDF 33 kb)

Additional file 21: *Agave americana* transcript sequences of less than 200 bp. (FASTA 5010 kb)

Additional file 22: Representative protein sequences in *Agave americana*. (FASTA 9640 kb)

Additional file 23: Representative protein sequences in *Agave deserti*. (FASTA 11600 kb)

Additional file 24: Representative protein sequences in *Agave tequilana*. (FASTA 11400 kb)

Additional file 25: Expression data of *Agave americana* transcripts detected in at least 4 of the 15 samples, with an average expression level of 5 RPKM or higher. (XLSX 14100 kb)

## Abbreviations

CAM: Crassulacean acid metabolism
CCA1: Circadian clock associated 1
CT-BMY: Chloroplast beta-amylase
ELF3: Early flowering 3
FUBAR: Fast unconstrained bayesian approximation
I-TASSER: Iterative threading assembly refinement
LHY: Late elongated hypocotyl
LUX: Lux arrhythmo
NVP: Non-vascular plant
PEPC: Phosphoenolpyruvate carboxylase.
PHOT2: Phototropin-2
PPCK1: phosphoenolpyruvate carboxylase kinase 1
RBH: Reciprocal best hits
REL: Random effects likelihood
RuBisCO: Ribulose-1,5-bisphosphate carboxylase/oxygenase
RVE1: Reveille 1
TOC1: Timing of cab expression1
