# Correction to: Diel rewiring and positive selection of ancient plant proteins enabled evolution of CAM photosynthesis in *Agave*

**DOI:** 10.1186/s12864-019-5663-8

**Published:** 2019-04-10

**Authors:** Hengfu Yin, Hao-Bo Guo, David J. Weston, Anne M. Borland, Priya Ranjan, Paul E. Abraham, Sara S. Jawdy, James Wachira, Gerald A. Tuskan, Timothy J. Tschaplinski, Stan D. Wullschleger, Hong Guo, Robert L. Hettich, Stephen M. Gross, Zhong Wang, Axel Visel, Xiaohan Yang

**Affiliations:** 1 Biosciences Division, Oak Ridge National Laboratory, Oak Ridge, TN 37831 USA; 2 Department of Biochemistry & Cellular and Molecular Biology, University of Tennessee, Knoxville, TN 37996 USA; 3 School of Natural and Environmental Sciences, Newcastle University, Newcastle upon Tyne, NE1 7RU UK; 4 DOE-Center for Bioenergy Innovation (CBI), Oak Ridge National Laboratory, Oak Ridge, TN 37831 USA; 5 Chemical Sciences Division, Oak Ridge National Laboratory, Oak Ridge, TN 37831 USA; 6 Department of Biology, Morgan State University, Baltimore, MD 21251 USA; 7 Environmental Sciences Division, Oak Ridge National Laboratory, Oak Ridge, TN 37831 USA; 8 DOE Joint Genome Institute, Walnut Creek, CA 94598 USA; 9 School of Natural Sciences, University of California, Merced, CA 95343 USA; 10 Environmental Genomics and Systems Biology Division, Lawrence Berkeley National Laboratory, Berkeley, CA 94720 USA; 11 Present address: Research Institute of Subtropical Forestry, Chinese Academy of Forestry, Hangzhou, 311400 Zhejiang China; 12 Present address: Illumina, Inc., San Diego, CA 92122 USA

**Correction to: Yin et al. BMC Genomics**

**https://doi.org/10.1186/s12864-018-4964-7**

Following publication of the original article [1], the authors found that the “Table S2” was redundantly put in both “Additional file 2” and “Additional file 3”, while “Table S1. Percentage of the gene set in each individual species distributed into different ortholog clades” was missed during the process of splitting a single combined supplementary file into individual files. A corrected version of “Additional file 2” is published in this Correction article.

## Additional file

Additional file 2: **Table S1.** Percentage of the gene set in each individual species distributed into different ortholog clades. (DOCX 20 kb)
