# Supplementary material for: Diel rewiring and positive selection of ancient plant proteins enabled evolution of CAM photosynthesis in Agave
Source: BMC Genomics. 2018 Aug 6;19:588. doi: 10.1186/s12864-018-4964-7 (PMC6090859; doi:10.1186/s12864-018-4964-7)
Supplement: Supplementary file 1 — Figure S1. Overview charts of the gene ontology (GO) biological processes over-represented in individual co-expression modules. (PDF 464 kb) [file 12864_2018_4964_MOESM1_ESM.pdf]

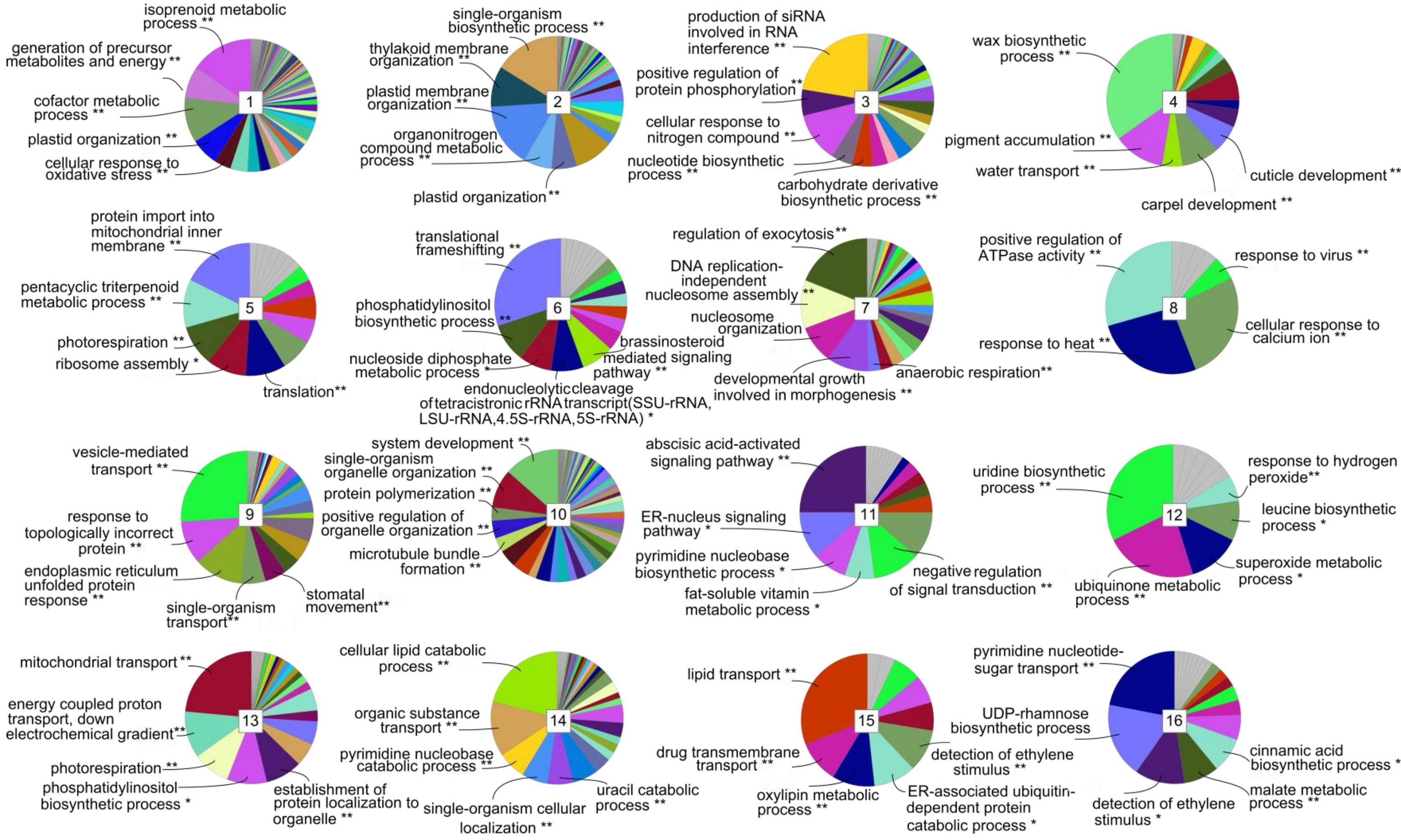

Figure S1. Overview charts of the gene ontology (GO) biological processes over-represented in individual co-expression modules. Only the names of the top 5 GO processes are shown. The number in the center of the pie charts represents the individual modules. For example, “1” and “11” represent modules M01and M11, respectively. Each process is represented by the group leading GO term (i.e., the most significant term in the group). The size of the group sections correlates with the number of the GO terms included in each group. “*” and “**” indicate that the GO process is over-represented at p-value < 0.05 and p-value <0.001, respectively. In each pie chart, GO processes with more than one member are represented by different colors. A GO process that did not group is represented as grey. Note: Colors do not correlate across overview charts.
