# Supplementary material for: Diel rewiring and positive selection of ancient plant proteins enabled evolution of CAM photosynthesis in Agave
Source: BMC Genomics. 2018 Aug 6;19:588. doi: 10.1186/s12864-018-4964-7 (PMC6090859; doi:10.1186/s12864-018-4964-7)
Supplement: Supplementary file 2 — Table S1. Percentage of the gene set in each individual species distributed into different ortholog clades. (PDF 39 kb) [file 12864_2018_4964_MOESM2_ESM.pdf]

Table S2. Biological processes over-represented ($p<1E-20$) in ortholog clade NVP:C_3:CAM:C_4 in *Agave americana*. NVP:C_3:CAM:C_4 represents orthologs shared by C_3, CAM, C_4 and NVP (i.e. non-vascular plants).

GO ID	Corrected p-value	Description
44237	3.44E-73	cellular metabolic process
44238	2.20E-69	primary metabolic process
8152	6.80E-66	metabolic process
44281	9.20E-62	small molecule metabolic process
9987	3.38E-55	cellular process
44249	5.94E-51	cellular biosynthetic process
9058	2.61E-48	biosynthetic process
9056	5.41E-42	catabolic process
19538	2.12E-36	protein metabolic process
44262	2.44E-33	cellular carbohydrate metabolic process
44267	6.01E-33	cellular protein metabolic process
5975	6.01E-33	carbohydrate metabolic process
16043	3.23E-30	cellular component organization
44260	4.12E-30	cellular macromolecule metabolic process
44248	5.47E-30	cellular catabolic process
34641	7.98E-30	cellular nitrogen compound metabolic process
43170	6.66E-29	macromolecule metabolic process
44282	1.34E-28	small molecule catabolic process
6807	1.80E-28	nitrogen compound metabolic process
6066	1.95E-27	alcohol metabolic process
5996	1.52E-25	monosaccharide metabolic process
46483	1.98E-24	heterocycle metabolic process
9059	3.14E-24	macromolecule biosynthetic process
34645	6.80E-23	cellular macromolecule biosynthetic process
6996	1.30E-22	organelle organization
55086	1.67E-22	nucleobase, nucleoside and nucleotide metabolic process
9117	4.70E-22	nucleotide metabolic process
6753	4.70E-22	nucleoside phosphate metabolic process
44085	8.25E-22	cellular component biogenesis
42180	2.22E-21	cellular ketone metabolic process
