# Supplementary material for: Diel rewiring and positive selection of ancient plant proteins enabled evolution of CAM photosynthesis in Agave
Source: BMC Genomics. 2018 Aug 6;19:588. doi: 10.1186/s12864-018-4964-7 (PMC6090859; doi:10.1186/s12864-018-4964-7)
Supplement: Supplementary file 4 — Table S3. Biological processes over-represented in ortholog clade C3:CAM:C4 in Agave americana. C3:CAM:C4 represents orthologs shared only by C3, CAM and C4 species. (PDF 17 kb) [file 12864_2018_4964_MOESM4_ESM.pdf]

Table S3. Biological processes over-represented in ortholog clade C_3:CAM:C_4 in *Agave americana*. C_3:CAM:C_4 represents orthologs shared only by C_3, CAM and C_4 species.

GO ID	Corrected	Description
9963	6.93E-06	positive regulation of flavonoid biosynthetic process
45449	5.65E-05	regulation of transcription
9962	6.42E-05	regulation of flavonoid biosynthetic process
6355	6.42E-05	regulation of transcription, DNA-dependent
51252	8.74E-05	regulation of RNA metabolic process
9889	8.74E-05	regulation of biosynthetic process
31326	8.74E-05	regulation of cellular biosynthetic process
10200	2.44E-04	response to chitin
10468	2.59E-04	regulation of gene expression
80090	5.23E-04	regulation of primary metabolic process
10089	6.52E-04	xylem development
10556	6.52E-04	regulation of macromolecule biosynthetic process
6855	6.97E-04	drug transmembrane transport
15893	6.97E-04	drug transport
19219	6.97E-04	regulation of nucleobase, nucleoside, nucleotide and nucleic acid metabolic process
31323	9.84E-04	regulation of cellular metabolic process
60255	9.84E-04	regulation of macromolecule metabolic process
42493	9.84E-04	response to drug
51171	1.25E-03	regulation of nitrogen compound metabolic process
19222	1.33E-03	regulation of metabolic process
10087	2.22E-03	phloem or xylem histogenesis
43455	5.07E-03	regulation of secondary metabolic process
9725	2.72E-02	response to hormone stimulus
9061	4.01E-02	anaerobic respiration
