# Supplementary material for: Diel rewiring and positive selection of ancient plant proteins enabled evolution of CAM photosynthesis in Agave
Source: BMC Genomics. 2018 Aug 6;19:588. doi: 10.1186/s12864-018-4964-7 (PMC6090859; doi:10.1186/s12864-018-4964-7)
Supplement: Supplementary file 5 — Table S4. Ortholog clades of C4 cycle genes & related transporters. (PDF 118 kb) [file 12864_2018_4964_MOESM5_ESM.pdf]

Table S4. Ortholog clades of C_4 cycle genes & related transporters.

Arabidopsis gene*	*Zea mays* gene	Name	Pathway	Ortholog clade**
AT1G17290	GRMZM2G053999	AlaAT	C4 cycle	NVP:C_3:CAM:C_4
AT2G22250	GRMZM2G033799	AspAT	C4 cycle	NVP:C_3:CAM:C_4
AT4G31990	GRMZM5G836910	AspAT	C4 cycle	NVP:C_3:CAM:C_4
AT1G62800	GRMZM2G094712	AspAT	C4 cycle	NVP:C_3:CAM:C_4
AT5G19550	GRMZM2G094712	AspAT	C4 cycle	NVP:C_3:CAM:C_4
AT5G11520	GRMZM2G094712	AspAT	C4 cycle	NVP:C_3:CAM:C_4
AT2G30970	GRMZM2G146677	AspAT	C4 cycle	NVP:C_3:CAM:C_4
AT3G47520	GRMZM2G068455	cpNAD-DH	C4 cycle	NVP:C_3:CAM:C_4
AT5G58330	GRMZM2G129513	cpNADP-DH	C4 cycle	NVP:C_3:CAM:C_4
AT1G53240	GRMZM2G154595	mtNAD-DH	C4 cycle	NVP:C_3:CAM:C_4
AT4G00570	GRMZM2G085747	NAD_ME	C4 cycle	NVP:C_3:CAM:C_4
AT2G13560	GRMZM2G085747	NAD_ME	C4 cycle	NVP:C_3:CAM:C_4
AT1G79750	GRMZM2G085019	NADP-E	C4 cycle	NVP:C_3:CAM:C_4
AT2G19900	GRMZM2G085019	NADP-E	C4 cycle	NVP:C_3:CAM:C_4
AT5G11670	GRMZM2G085019	NADP-E	C4 cycle	NVP:C_3:CAM:C_4
AT1G68750	GRMZM2G082780	PEPC	C4 cycle	NVP:C_3:CAM:C_4
AT1G53310	GRMZM2G069542	PEPC	C4 cycle	NVP:C_3:CAM:C_4
AT2G42600	GRMZM2G069542	PEPC	C4 cycle	NVP:C_3:CAM:C_4
AT5G65690	GRMZM2G001696	PEP-CK	C4 cycle	NVP:C_3:CAM:C_4
AT4G37870	GRMZM2G001696	PEP-CK	C4 cycle	NVP:C_3:CAM:C_4
AT2G30130	GRMZM2G044150	PEP-CK	C4 cycle	NVP:C_3:CAM:C_4
AT4G15530	GRMZM2G097457	PPDK	C4 cycle	NVP:C_3:CAM:C_4
AT5G35170	GRMZM2G030628	adenylate kinase family protein	processing of PPDK products	NVP:C_3:CAM:C_4
AT5G47840	GRMZM2G178192	AMK2	processing of PPDK products	NVP:C_3:CAM:C_4
AT5G09650	GRMZM2G090718	PPA	processing of PPDK products	NVP:C_3:CAM:C_4
AT3G04530	GRMZM2G178074	PEPC-K	regulatory proteins	C_3:CAM:C_4
AT1G08650	GRMZM2G049541	PEPC-K	regulatory proteins	C_3:CAM:C_4
AT4G21210	GRMZM2G004880	PPDK-RP	regulatory proteins	NVP:C_3:CAM:C_4
AT3G56160	GRMZM2G092475	BASS 4	transport	NVP:C_3:CAM:C_4
AT5G12860	GRMZM2G383088	Dit1	transport	NVP:C_3:CAM:C_4
AT5G64280	GRMZM2G040933	Dit2	transport	NVP:C_3:CAM:C_4
AT1G49810	GRMZM2G053927	NHD	transport	NVP:C_3:CAM:C_4
AT5G33320	GRMZM2G047404	PPT1	transport	NVP:C_3:CAM:C_4
AT5G46110	GRMZM2G070605	TPT	transport	NVP:C_3:CAM:C_4

*Gene names were obtained from Gowik *et al*. (2011).

**NVP:C_3:CAM:C_4 represents orthologs shared by NVP, C_3, CAM and C_4. C_3:CAM:C_4 represents orthologs shared only by C_3, CAM and C_4.
