# Supplementary material for: Diel rewiring and positive selection of ancient plant proteins enabled evolution of CAM photosynthesis in Agave
Source: BMC Genomics. 2018 Aug 6;19:588. doi: 10.1186/s12864-018-4964-7 (PMC6090859; doi:10.1186/s12864-018-4964-7)
Supplement: Supplementary file 6 — Table S5. Distribution of co-expression modules in each individual ortholog clade in Agave americana. (PDF 90 kb) [file 12864_2018_4964_MOESM6_ESM.pdf]

Table S5. Distribution of co-expression modules in each individual ortholog clade in *Agave americana*. The numbers represent the observed and expected (in parentheses) number of genes in each pairwise combination of co-expression modules and ortholog clades.

Module	NVP:C_3:CAM:C_4	NVP:C_3:CAM	NVP:CAM:C_4	NVP:CAM	C_3:CAM:C_4	C_3:CAM	CAM:C_4	CAM-only	Total
M01	1796 (1543)[a]	34 (34)	0 (1)	3 (11)	318 (409)[b]	60 (96)[b]	11 (10)	213 (330)[b]	2435
M02	404 (393)	15 (9)	0 (0)	1 (3)	87 (104)	28 (25)	3 (3)	82 (84)	620
M03	419 (414)	12 (9)	0 (0)	4 (3)	107 (110)	25 (26)	2 (3)	85 (89)	654
M04	201 (278)[b]	12 (6)	0 (0)	2 (2)	113 (74)[a]	19 (17)	1 (2)	90 (59)[a]	438
M05	251 (215)	1 (5)	0 (0)	0 (2)	38 (57)	7 (13)	1 (1)	42 (46)	340
M06	215 (217)	1 (5)	0 (0)	1 (2)	63 (57)	16 (14)	0 (1)	46 (46)	342
M07	520 (580)	8 (13)	1 (0)	7 (4)	189 (154)[a]	40 (36)	3 (4)	147 (124)	915
M08	90 (112)	1 (2)	0 (0)	2 (1)	47 (30)[a]	6 (7)	0 (1)	31 (24)	177
M09	466 (521)	12 (12)	1 (0)	8 (4)	160 (138)	39 (33)	1 (4)	136 (112)	823
M10	1434 (1547)[b]	47 (34)	1 (1)	10 (11)	404 (410)	104 (97)	11 (10)	430 (331)[a]	2441
M11	200 (272)[b]	5 (6)	0 (0)	4 (2)	123 (72)[a]	24 (17)	1 (2)	72 (58)	429
M12	113 (151)[b]	1 (3)	0 (0)	2 (1)	47 (40)	19 (9)	0 (1)	56 (32)[a]	238
M13	592 (603)	10 (13)	1 (0)	3 (4)	163 (160)	50 (38)	5 (4)	127 (129)	951
M14	457 (471)	10 (10)	0 (0)	8 (3)	150 (125)	24 (29)	3 (3)	92 (101)	744
M15	78 (103)	1 (2)	0 (0)	1 (1)	38 (27)	14 (6)	3 (1)	27 (22)	162
M16	122 (136)	3 (3)	0 (0)	1 (1)	39 (36)	7 (9)	1 (1)	42 (29)	215
other	1629 (1432)[a]	27 (32)	1 (1)	9 (11)	295 (379)[b]	79 (89)	15 (10)	205 (306)[b]	2260

[a]Overrepresentation ($P < 0.01$, cumulative Poisson distribution) of co-expression modules in each ortholog clade. [b]Underrepresentation ($P < 0.01$, cumulative Poisson distribution) of co-expression modules in each ortholog clade.

Note: NVP:C_3:CAM:C_4 represents orthologs shared by NVP, C_3, CAM and C_4 (i.e. non-vascular plants). NVP:C_3:CAM represents orthologs shared only by NVP, C_3 and CAM. NVP:CAM:C_4 represents orthologs shared only by NVP, CAM and C_4. NVP:CAM represents orthologs shared only by NVP and CAM. C_3:CAM:C_4 represents orthologs shared only by C_3, CAM and C_4. C_3:CAM represents orthologs shared only by C_3 and CAM. CAM:C_4 represents orthologs shared only by CAM and C_4. CAM-only represent orthologs only in CAM species.
