# Supplementary material for: Diel rewiring and positive selection of ancient plant proteins enabled evolution of CAM photosynthesis in Agave
Source: BMC Genomics. 2018 Aug 6;19:588. doi: 10.1186/s12864-018-4964-7 (PMC6090859; doi:10.1186/s12864-018-4964-7)
Supplement: Supplementary file 7 — Table S6. List of Agave americana genes that have homologs in other three independent CAM lineages but not in 21 non-CAM plant species. (PDF 31 kb) [file 12864_2018_4964_MOESM7_ESM.pdf]

Table S6. List of *Agave americana* genes that have homologs in other three independent CAM lineages but not in 21 non-CAM plant species.

**Gene ID**	**Functional description**
Aam366668	Unknown
Aam074797	Unknown
Aam052521	light-regulated zinc finger protein, B-box domain protein
Aam052525	light-regulated zinc finger protein, B-box domain protein
Aam052527	light-regulated zinc finger protein, B-box domain protein
Aam344359	light-regulated zinc finger protein, B-box domain protein
Aam301242	Unknown
Aam073892	nucleolin like protein
Aam304970	nuclear factor Y like protein
Aam213899	CLAVATA3/ESR-RELATED protein
Aam009930	global transcription factor group E7 protein
Aam047295	GATA transcription factor 11 like protein
Aam082065	histone deacetylase like
