# Supplementary material for: Diel rewiring and positive selection of ancient plant proteins enabled evolution of CAM photosynthesis in Agave
Source: BMC Genomics. 2018 Aug 6;19:588. doi: 10.1186/s12864-018-4964-7 (PMC6090859; doi:10.1186/s12864-018-4964-7)
Supplement: Supplementary file 8 — Figure S2. The diel expression pattern of conserved CAM-specific genes in Agave. (PDF 111 kb) [file 12864_2018_4964_MOESM8_ESM.pdf]

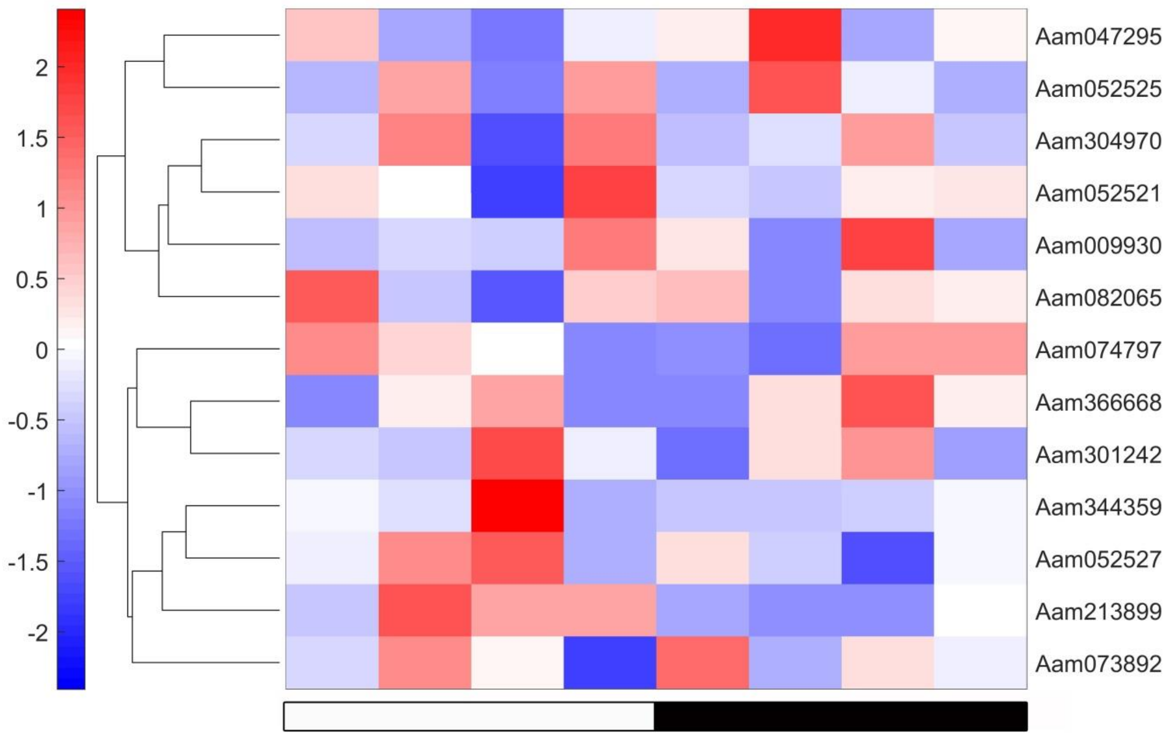

Figure S2. The diel expression pattern of conserved CAM-specific genes in *Agave*. The expression levels were normalized by z-score and visualized by heatmap plot. White and black bars indicate daytime (12-hour) and nighttime (12-hour), respectively.
