# Supplementary material for: Diel rewiring and positive selection of ancient plant proteins enabled evolution of CAM photosynthesis in Agave
Source: BMC Genomics. 2018 Aug 6;19:588. doi: 10.1186/s12864-018-4964-7 (PMC6090859; doi:10.1186/s12864-018-4964-7)
Supplement: Supplementary file 9 — Table S7. List of Agave americana genes with Ka/Ks ratio greater than one in Agave-Arabidopsis, Agave-Oryza and Agave-Zea pairs. (PDF 353 kb) [file 12864_2018_4964_MOESM9_ESM.pdf]

Table S7. List of *Agave americana* genes with Ka/Ks ratio greater than one in *Agave-Arabidopsis*, *Agave-Oryza* and *Agave-Zea* pairs. P-values indicating the significance of Ka/Ks ratio greater than one, as calculated using one-sided t-test. “nd” indicates not enough positive sites for significance tests.

Gene ID	Ortholog clade	P-value (*Agave-Arabidopsis*)	P-value (*Agave-Oryza*)	P-value (*Agave-Zea*)
**Aam00023**	C3:CAM:C4	1.00E-04	1.57E-07	5.10E-08
**Aam04413**	C3:CAM:C4	nd	2.03E-24	6.11E-23
**Aam04795**	C3:CAM:C4	1.18E-02	6.59E-23	1.49E-25
**Aam07788**	C3:CAM:C4	nd	5.40E-52	1.86E-24
**Aam01341**	C3:CAM:C4	6.82E-09	2.59E-36	1.35E-50
**Aam04567**	C3:CAM:C4	1.60E-15	3.38E-71	5.71E-42
**Aam00473**	C3:CAM:C4	9.34E-02	3.31E-43	4.56E-56
**Aam04553**	C3:CAM:C4	1.27E-10	1.22E-39	9.85E-30
**Aam08546**	C3:CAM:C4	1.90E-07	1.14E-26	9.75E-35
**Aam08778**	C3:CAM:C4	1.63E-02	6.51E-38	1.58E-25
**Aam08786**	C3:CAM:C4	3.72E-08	1.66E-80	6.99E-72
**Aam08840**	C3:CAM:C4	1.28E-28	4.90E-93	2.63E-50
**Aam04352**	C3:CAM:C4	1.20E-03	4.49E-32	4.16E-84
**Aam00635**	C3:CAM:C4	1.32E-05	3.68E-25	3.52E-28
**Aam04808**	C3:CAM:C4	8.60E-02	1.06E-35	1.70E-109
**Aam00062**	C3:CAM:C4	1.25E-04	7.63E-60	1.07E-26
**Aam01359**	C3:CAM:C4	8.94E-08	7.88E-29	6.51E-31
**Aam04423**	C3:CAM:C4	nd	2.26E-21	3.98E-23
**Aam05001**	C3:CAM:C4	nd	2.39E-63	7.46E-95
**Aam07677**	C3:CAM:C4	2.53E-03	3.09E-10	9.64E-07
**Aam08294**	C3:CAM:C4	5.60E-02	2.96E-43	1.00E-06
**Aam08295**	C3:CAM:C4	4.83E-08	1.38E-12	2.88E-36
**Aam08470**	C3:CAM:C4	4.39E-04	2.14E-20	2.62E-11
**Aam08603**	C3:CAM:C4	9.53E-04	4.98E-78	9.75E-35
**Aam08644**	C3:CAM:C4	8.55E-03	9.45E-02	3.01E-39
**Aam08710**	C3:CAM:C4	3.61E-09	1.07E-24	6.85E-55
**Aam08734**	C3:CAM:C4	6.45E-07	4.92E-64	6.81E-45
**Aam08754**	C3:CAM:C4	1.60E-02	3.81E-28	2.70E-17
**Aam08769**	C3:CAM:C4	7.13E-14	2.18E-50	6.12E-93
**Aam08821**	C3:CAM:C4	3.13E-26	1.06E-104	5.81E-67
**Aam08831**	C3:CAM:C4	6.48E-11	2.49E-18	7.69E-49
**Aam09352**	C3:CAM:C4	1.31E-02	1.87E-24	5.89E-05
**Aam34102**	C3:CAM:C4	nd	1.95E-26	7.38E-21
**Aam34501**	C3:CAM:C4	3.60E-03	6.42E-26	2.17E-28
**Aam07856**	C3:CAM:C4	2.52E-02	2.50E-43	5.24E-34
**Aam07553**	C3:CAM:C4	2.60E-02	1.03E-01	nd

**Aam00373**	C3:CAM:C4	1.27E-15	7.49E-07	4.77E-14
**Aam00252**	C3:CAM:C4	8.07E-03	3.55E-36	4.32E-32
**Aam01365**	C3:CAM:C4	1.95E-02	9.06E-98	2.16E-63
**Aam04498**	C3:CAM:C4	4.39E-04	2.06E-20	3.95E-49
**Aam05516**	NVP:C3:CAM:C4	1.49E-08	1.10E-41	2.35E-43
**Aam05793**	NVP:C3:CAM:C4	2.68E-02	1.69E-56	2.59E-60
**Aam08122**	NVP:C3:CAM:C4	3.31E-02	1.63E-15	5.47E-36
**Aam08663**	NVP:C3:CAM:C4	1.16E-26	5.98E-37	2.55E-49
**Aam08718**	NVP:C3:CAM:C4	3.71E-25	1.61E-70	1.01E-20
**Aam08782**	NVP:C3:CAM:C4	6.23E-08	3.93E-34	8.42E-46
**Aam34310**	NVP:C3:CAM:C4	2.41E-07	5.13E-15	1.23E-32
**Aam01284**	NVP:C3:CAM:C4	8.48E-02	3.12E-44	1.73E-43
**Aam04474**	NVP:C3:CAM:C4	1.00E-01	2.96E-37	1.66E-29
**Aam04431**	NVP:C3:CAM:C4	5.26E-09	1.12E-14	3.33E-21
**Aam05128**	NVP:C3:CAM:C4	8.19E-07	3.16E-36	1.38E-07
**Aam08135**	NVP:C3:CAM:C4	3.08E-04	2.27E-50	2.69E-30
**Aam08506**	NVP:C3:CAM:C4	nd	1.10E-72	2.88E-37
**Aam08000**	NVP:C3:CAM:C4	7.78E-03	5.65E-51	4.14E-55
**Aam00023**	NVP:C3:CAM:C4	8.06E-10	2.67E-71	2.21E-51
**Aam00468**	NVP:C3:CAM:C4	1.11E-07	3.57E-61	1.59E-45
**Aam01303**	NVP:C3:CAM:C4	1.55E-10	1.11E-93	1.58E-74
**Aam01357**	NVP:C3:CAM:C4	1.64E-01	5.46E-33	3.24E-65
**Aam01364**	NVP:C3:CAM:C4	3.02E-02	1.18E-36	1.08E-43
**Aam04585**	NVP:C3:CAM:C4	7.08E-03	4.07E-60	1.69E-52
**Aam05327**	NVP:C3:CAM:C4	8.88E-02	2.11E-37	1.05E-46
**Aam08025**	NVP:C3:CAM:C4	2.01E-01	2.00E-23	3.70E-03
**Aam08051**	NVP:C3:CAM:C4	8.16E-03	9.06E-65	1.90E-65
**Aam08205**	NVP:C3:CAM:C4	1.87E-09	1.68E-35	5.99E-42
**Aam08234**	NVP:C3:CAM:C4	3.20E-07	1.53E-23	1.32E-19
**Aam08283**	NVP:C3:CAM:C4	2.27E-08	1.70E-69	8.70E-67
**Aam08325**	NVP:C3:CAM:C4	1.59E-07	6.83E-111	4.17E-90
**Aam08328**	NVP:C3:CAM:C4	1.99E-02	4.83E-40	5.76E-76
**Aam08521**	NVP:C3:CAM:C4	3.86E-18	4.36E-107	5.70E-66
**Aam08581**	NVP:C3:CAM:C4	1.31E-14	5.74E-84	5.18E-47
**Aam08681**	NVP:C3:CAM:C4	4.66E-05	7.44E-100	1.06E-48
**Aam08740**	NVP:C3:CAM:C4	3.39E-07	6.61E-43	1.55E-27
**Aam08742**	NVP:C3:CAM:C4	5.71E-25	2.84E-35	6.44E-38
**Aam08747**	NVP:C3:CAM:C4	5.09E-03	2.79E-32	9.51E-07
**Aam08758**	NVP:C3:CAM:C4	1.67E-11	6.94E-49	1.76E-49
**Aam08775**	NVP:C3:CAM:C4	3.18E-14	5.25E-67	5.88E-77
**Aam08781**	NVP:C3:CAM:C4	2.15E-14	3.47E-40	3.14E-45
**Aam08806**	NVP:C3:CAM:C4	1.05E-21	5.95E-140	7.44E-103
**Aam08814**	NVP:C3:CAM:C4	2.22E-05	5.20E-34	4.69E-13
**Aam08836**	NVP:C3:CAM:C4	8.67E-17	1.24E-54	4.09E-32

**Aam04507**	C3:CAM:C4	1.57E-06	8.86E-18	1.73E-10
**Aam04509**	C3:CAM:C4	8.77E-26	5.77E-89	1.14E-91
**Aam04542**	C3:CAM:C4	2.84E-04	5.99E-06	4.60E-35
**Aam04586**	C3:CAM:C4	1.20E-04	8.80E-58	1.00E-58
**Aam04587**	C3:CAM:C4	5.63E-02	2.09E-47	2.99E-84
**Aam05283**	C3:CAM:C4	2.40E-02	1.78E-18	3.56E-16
**Aam07933**	C3:CAM:C4	2.18E-02	4.98E-56	1.47E-91
**Aam08352**	C3:CAM:C4	1.93E-06	2.08E-09	5.47E-24
**Aam08449**	C3:CAM:C4	nd	2.29E-44	2.60E-30
**Aam08609**	C3:CAM:C4	1.16E-12	1.04E-45	9.14E-28
**Aam08782**	C3:CAM:C4	4.11E-09	4.51E-39	1.46E-39
**Aam08788**	C3:CAM:C4	1.86E-03	2.33E-54	1.55E-45
**Aam08798**	C3:CAM:C4	2.46E-03	2.62E-24	1.33E-23
**Aam08828**	C3:CAM:C4	9.84E-19	1.96E-48	1.66E-40
**Aam08831**	C3:CAM:C4	1.31E-08	1.74E-60	9.62E-68
**Aam09416**	C3:CAM:C4	1.29E-24	3.05E-54	3.17E-30
**Aam00048**	NVP:C3:CAM:C4	5.98E-05	1.75E-44	2.53E-49
**Aam00918**	NVP:C3:CAM:C4	3.86E-07	6.31E-18	3.14E-10
**Aam01800**	NVP:C3:CAM:C4	2.43E-15	2.10E-37	2.28E-44
**Aam01857**	NVP:C3:CAM:C4	nd	1.51E-16	8.72E-21
**Aam04466**	NVP:C3:CAM:C4	8.93E-08	5.74E-71	6.38E-56
**Aam04875**	NVP:C3:CAM:C4	1.55E-01	1.66E-09	4.09E-32
**Aam05101**	NVP:C3:CAM:C4	5.56E-05	4.65E-23	1.79E-57
**Aam05587**	NVP:C3:CAM:C4	4.19E-07	7.31E-34	1.78E-32
**Aam08286**	NVP:C3:CAM:C4	6.86E-13	nd	0.00E+00
**Aam08442**	NVP:C3:CAM:C4	2.32E-05	7.33E-95	2.90E-58
**Aam04505**	NVP:C3:CAM:C4	2.10E-14	9.25E-86	3.56E-55
**Aam04565**	NVP:C3:CAM:C4	3.45E-15	8.55E-42	9.04E-50
**Aam07799**	NVP:C3:CAM:C4	2.79E-08	1.38E-54	6.81E-32
**Aam07894**	NVP:C3:CAM:C4	2.26E-07	2.31E-79	9.84E-35
**Aam07901**	NVP:C3:CAM:C4	nd	2.58E-102	9.16E-61
**Aam07926**	NVP:C3:CAM:C4	2.98E-02	5.55E-23	2.42E-21
**Aam08204**	NVP:C3:CAM:C4	1.36E-08	2.86E-25	2.18E-38
**Aam08497**	NVP:C3:CAM:C4	nd	3.10E-39	2.81E-66
**Aam08598**	NVP:C3:CAM:C4	1.23E-02	2.94E-46	7.37E-72
**Aam08711**	NVP:C3:CAM:C4	1.07E-03	6.95E-114	1.80E-80
**Aam08814**	NVP:C3:CAM:C4	2.32E-03	7.02E-51	1.30E-68
**Aam00476**	NVP:C3:CAM:C4	5.75E-02	3.58E-14	2.20E-24
**Aam01025**	NVP:C3:CAM:C4	1.41E-05	3.69E-19	2.73E-37
**Aam04971**	NVP:C3:CAM:C4	1.86E-11	2.21E-21	4.43E-18
**Aam34482**	NVP:C3:CAM:C4	1.44E-05	4.14E-52	4.33E-27
**Aam34534**	NVP:C3:CAM:C4	2.40E-04	2.10E-18	1.37E-08
**Aam34963**	NVP:C3:CAM:C4	3.07E-05	3.25E-08	1.10E-17
**Aam35460**	NVP:C3:CAM:C4	5.17E-10	1.93E-69	3.06E-61

**Aam01159**	NVP:C3:CAM:C4	3.30E-02	1.83E-33	2.06E-07
**Aam04932**	NVP:C3:CAM:C4	1.94E-01	6.58E-67	6.41E-31
**Aam08544**	NVP:C3:CAM:C4	1.38E-03	6.62E-48	1.51E-52
**Aam34148**	NVP:C3:CAM:C4	2.76E-06	3.23E-18	4.33E-21
**Aam05438**	NVP:C3:CAM:C4	1.64E-10	4.12E-12	9.56E-13
**Aam08176**	NVP:C3:CAM:C4	1.59E-01	1.08E-38	4.59E-38
**Aam08787**	NVP:C3:CAM:C4	3.99E-03	1.88E-88	2.42E-63
**Aam08157**	NVP:C3:CAM:C4	1.47E-01	2.79E-23	1.23E-22
**Aam00477**	NVP:C3:CAM:C4	2.11E-15	2.32E-65	3.46E-99
**Aam01020**	NVP:C3:CAM:C4	6.36E-08	3.62E-71	9.82E-58
**Aam04356**	NVP:C3:CAM:C4	4.28E-08	4.05E-06	2.79E-20
**Aam04378**	NVP:C3:CAM:C4	1.73E-10	1.76E-26	1.12E-45
**Aam04386**	NVP:C3:CAM:C4	2.12E-02	1.39E-32	4.40E-25
**Aam04520**	NVP:C3:CAM:C4	9.01E-06	3.54E-68	8.64E-21
**Aam04579**	NVP:C3:CAM:C4	1.07E-06	2.61E-31	4.22E-42
**Aam04580**	NVP:C3:CAM:C4	3.25E-11	5.66E-32	3.06E-48
**Aam04585**	NVP:C3:CAM:C4	3.33E-09	7.08E-103	9.68E-109
**Aam04589**	NVP:C3:CAM:C4	6.02E-15	8.89E-135	7.70E-126
**Aam04593**	NVP:C3:CAM:C4	4.78E-04	6.42E-54	5.86E-88
**Aam05253**	NVP:C3:CAM:C4	nd	1.78E-23	1.96E-06
**Aam05414**	NVP:C3:CAM:C4	4.09E-03	2.05E-41	3.84E-63
**Aam05729**	NVP:C3:CAM:C4	1.23E-03	9.51E-30	5.19E-57
**Aam07884**	NVP:C3:CAM:C4	3.72E-03	4.18E-24	1.34E-31
**Aam08264**	NVP:C3:CAM:C4	2.95E-03	1.09E-60	1.47E-25
**Aam08349**	NVP:C3:CAM:C4	2.74E-06	2.40E-90	1.11E-42
**Aam08512**	NVP:C3:CAM:C4	2.77E-10	3.66E-26	5.13E-29
**Aam08601**	NVP:C3:CAM:C4	4.35E-12	3.42E-28	1.34E-43
**Aam08724**	NVP:C3:CAM:C4	4.97E-10	2.00E-60	4.15E-19
**Aam08769**	NVP:C3:CAM:C4	2.09E-14	9.41E-30	1.90E-55
**Aam08770**	NVP:C3:CAM:C4	2.92E-06	2.43E-28	1.36E-40
**Aam08779**	NVP:C3:CAM:C4	5.44E-06	2.54E-20	2.38E-79
**Aam08802**	NVP:C3:CAM:C4	8.59E-07	1.19E-30	6.54E-32
**Aam08824**	NVP:C3:CAM:C4	1.06E-07	7.76E-100	2.79E-37
**Aam08834**	NVP:C3:CAM:C4	3.88E-03	2.18E-47	2.08E-108
**Aam08836**	NVP:C3:CAM:C4	3.62E-23	1.43E-70	3.73E-71
**Aam35294**	NVP:C3:CAM:C4	2.83E-02	1.14E-54	3.22E-37
