# Supplementary material for: Diel rewiring and positive selection of ancient plant proteins enabled evolution of CAM photosynthesis in Agave
Source: BMC Genomics. 2018 Aug 6;19:588. doi: 10.1186/s12864-018-4964-7 (PMC6090859; doi:10.1186/s12864-018-4964-7)
Supplement: Supplementary file 10 — Table S8. Genes implicated in the CAM pathway undergoing positive selection as revealed from Ka/Ks ratio calculated from Agave-Arabidopsis, Agave-Oryza and Agave-Zea orthologous gene pairs. (PDF 91 kb) [file 12864_2018_4964_MOESM10_ESM.pdf]

Table S8. Genes implicated in the CAM pathway undergoing positive selection as revealed from Ka/Ks ratio calculated from *Agave-Arabidopsis*, *Agave-Oryza* and *Agave-Zea* orthologous gene pairs.

**Agave gene**	**Name**	**Description**	**Function Category**	**Ortholog clade****
Aam048341[b]	PPCK1	Phosphoenolpyruvate carboxylase kinase 1	Carbon fixation	C_3:CAM:C_4
Aam048752[a]	PCL1 or LUX	PHYTOCLOCK 1	Circadian-core	NVP:C_3:CAM:C_4
Aam006353[a]	LHY	LATE ELONGATED HYPOCOTYL	Circadian-core	C_3:CAM:C_4
Aam085988[a]	ELF3	EARLY FLOWERING 3	Circadian-input	NVP:C_3:CAM:C_4
Aam051010[a]	PPDK-RP	PPDK regulatory protein	Decarboxylation light reaction	NVP:C_3:CAM:C_4
Aam002877[b]	PsI-D2	Photosystem I subunit D-2	Photosynthesis-PET	NVP:C_3:CAM:C_4
Aam088315[a]	GAUT7	Galacturonosyltransferase 7	Starch and sucrose metabolism	C_3:CAM:C_4
Aam082045[a]	SS2	Starch synthase 2	Starch and sucrose metabolism	NVP:C_3:CAM:C_4
Aam049326[a]	CT-BMY	Chloroplast beta-amylase	Starch and sucrose metabolism	NVP:C_3:CAM:C_4

*Note: "a" indicates Ka/Ks ratio greater than one in *Agave-Arabidopsis*, *Agave-Oryza* and *Agave-Zea* orthologous gene pair comparison. "b" indicates Ka/Ks ratio greater than one in *Agave-Arabidopsis* and *Agave-Oryza* and but not *Agave-Zea* orthologous gene pair comparison.

**NVP:C_3:CAM:C_4 represents orthologs shared by NVP, C_3, CAM and C_4. C_3:CAM:C_4 represents orthologs shared only by C_3, CAM and C_4.
