# Supplementary material for: Diel rewiring and positive selection of ancient plant proteins enabled evolution of CAM photosynthesis in Agave
Source: BMC Genomics. 2018 Aug 6;19:588. doi: 10.1186/s12864-018-4964-7 (PMC6090859; doi:10.1186/s12864-018-4964-7)
Supplement: Supplementary file 11 — Table S9. List of Agave americana genes with Ka/Ks ratio greater than one in Agave-Arabidopsis and Agave-Oryza but not Agave-Zea pairs. (PDF 47 kb) [file 12864_2018_4964_MOESM11_ESM.pdf]

Table S9. List of *Agave americana* genes with Ka/Ks ratio greater than one in *Agave-Arabidopsis* and *Agave-Oryza* but not *Agave-Zea* pairs.

Gene ID	Ortholog clade	Gene ID	Ortholog clade
Aam004683	C3:CAM:C4	Aam332970	NVP:C3:CAM:C4
Aam014899	C3:CAM:C4	Aam357923	NVP:C3:CAM:C4
Aam045646	C3:CAM:C4	Aam004469	NVP:C3:CAM:C4
Aam079585	C3:CAM:C4	Aam046130	NVP:C3:CAM:C4
Aam085954	C3:CAM:C4	Aam083292	NVP:C3:CAM:C4
Aam000508	C3:CAM:C4	Aam000891	NVP:C3:CAM:C4
Aam044860	C3:CAM:C4	Aam004408	NVP:C3:CAM:C4
Aam086287	C3:CAM:C4	Aam013663	NVP:C3:CAM:C4
Aam046915	C3:CAM:C4	Aam022194	NVP:C3:CAM:C4
Aam007696	C3:CAM:C4	Aam044505	NVP:C3:CAM:C4
Aam013071	C3:CAM:C4	Aam044597	NVP:C3:CAM:C4
Aam019908	C3:CAM:C4	Aam045648	NVP:C3:CAM:C4
Aam045853	C3:CAM:C4	Aam045861	NVP:C3:CAM:C4
Aam051597	C3:CAM:C4	Aam055741	NVP:C3:CAM:C4
Aam063676	C3:CAM:C4	Aam056318	NVP:C3:CAM:C4
Aam086603	C3:CAM:C4	Aam074009	NVP:C3:CAM:C4
Aam048341	C3:CAM:C4	Aam081496	NVP:C3:CAM:C4
Aam084654	C3:CAM:C4	Aam082363	NVP:C3:CAM:C4
Aam081889	C3:CAM:C4	Aam082779	NVP:C3:CAM:C4
Aam011021	C3:CAM:C4	Aam087015	NVP:C3:CAM:C4
Aam003350	C3:CAM:C4	Aam088209	NVP:C3:CAM:C4
Aam045814	C3:CAM:C4	Aam004640	NVP:C3:CAM:C4
Aam083376	C3:CAM:C4	Aam075912	NVP:C3:CAM:C4
Aam087989	C3:CAM:C4	Aam081307	NVP:C3:CAM:C4
Aam088045	C3:CAM:C4	Aam004340	NVP:C3:CAM:C4
Aam325436	C3:CAM:C4	Aam013411	NVP:C3:CAM:C4
Aam000258	NVP:C3:CAM:C4	Aam013450	NVP:C3:CAM:C4
Aam000764	NVP:C3:CAM:C4	Aam038631	NVP:C3:CAM:C4
Aam002877	NVP:C3:CAM:C4	Aam044051	NVP:C3:CAM:C4
Aam004269	NVP:C3:CAM:C4	Aam044297	NVP:C3:CAM:C4
Aam011027	NVP:C3:CAM:C4	Aam045742	NVP:C3:CAM:C4
Aam017081	NVP:C3:CAM:C4	Aam052293	NVP:C3:CAM:C4
Aam076244	NVP:C3:CAM:C4	Aam078199	NVP:C3:CAM:C4
Aam076551	NVP:C3:CAM:C4	Aam078452	NVP:C3:CAM:C4
Aam077266	NVP:C3:CAM:C4	Aam079965	NVP:C3:CAM:C4
Aam083451	NVP:C3:CAM:C4	Aam083978	NVP:C3:CAM:C4
Aam087428	NVP:C3:CAM:C4	Aam085207	NVP:C3:CAM:C4
Aam324992	NVP:C3:CAM:C4	Aam085507	NVP:C3:CAM:C4
Aam327516	NVP:C3:CAM:C4	Aam085920	NVP:C3:CAM:C4
Aam000286	NVP:C3:CAM:C4	Aam086219	NVP:C3:CAM:C4
Aam087017	NVP:C3:CAM:C4	Aam087037	NVP:C3:CAM:C4
Aam322360	NVP:C3:CAM:C4	Aam087461	NVP:C3:CAM:C4
Aam055616	NVP:C3:CAM:C4	Aam087490	NVP:C3:CAM:C4
Aam086482	NVP:C3:CAM:C4	Aam087513	NVP:C3:CAM:C4
Aam087837	NVP:C3:CAM:C4	Aam088035	NVP:C3:CAM:C4
Aam088368	NVP:C3:CAM:C4	Aam088351	NVP:C3:CAM:C4
Aam088382	NVP:C3:CAM:C4	Aam349697	NVP:C3:CAM:C4
