# Supplementary material for: Diel rewiring and positive selection of ancient plant proteins enabled evolution of CAM photosynthesis in Agave
Source: BMC Genomics. 2018 Aug 6;19:588. doi: 10.1186/s12864-018-4964-7 (PMC6090859; doi:10.1186/s12864-018-4964-7)
Supplement: Supplementary file 12 — Table S10. The positively selected sites of candidates with Ka/Ks > 1 from Table S9. (PDF 304 kb) [file 12864_2018_4964_MOESM12_ESM.pdf]

Table S10. The positively selected sites of candidates with Ka/Ks>1 from Table S9. The FUBAR model from HYPHY package were used to identify positively selected sites with posterior probabilities greater than 80%.

GeneID	Positive Codon	α	β	β-α	Posterior Prob β>α	Emp. Bayes Factor	PSRF	Neff
**Aam000258**	12	0.41	1.07	0.67	0.83	12.31	1.00	1190.36
**Aam000286**	17	0.78	2.89	2.10	0.84	17.40	1.00	553.82
	50	0.14	0.39	0.25	0.86	19.62	1.00	683.62
	552	0.60	1.80	1.20	0.82	14.56	1.00	671.85
**Aam000508**	28	0.26	0.70	0.44	0.81	13.58	1.00	3407.29
**Aam000764**	23	0.49	1.39	0.90	0.82	8.46	1.00	3198.77
	37	0.82	2.78	1.95	0.83	8.95	1.00	1729.78
	205	0.14	0.31	0.18	0.82	8.35	1.01	453.72
**Aam000891**	NO							
**Aam002877**	43	0.51	1.51	1.00	0.83	10.73	1.01	502.70
	60	0.74	2.70	1.96	0.83	10.65	1.00	675.17
**Aam003350**	NO							
**Aam004269**	NO							
**Aam004340**	23	0.96	3.08	2.12	0.81	15.91	1.00	1678.57
	73	1.33	4.42	3.09	0.80	15.11	1.00	1222.25
	75	0.84	3.51	2.67	0.87	25.36	1.00	2512.80
	92	0.25	0.78	0.53	0.83	18.35	1.00	1904.04
	539	1.09	4.16	3.07	0.83	17.71	1.00	1855.21
**Aam004408**	NO							
**Aam004469**	22	0.43	1.22	0.79	0.82	14.97	1.00	2014.72
**Aam004640**	319	0.23	0.43	0.20	0.82	8.96	1.00	1116.93
	339	0.98	2.94	1.96	0.81	8.37	1.00	1637.72
**Aam004683**	NO							
**Aam007696**	NO							
**Aam011021**	60	0.80	2.87	2.07	0.83	13.45	1.00	713.44
**Aam011027**	NO							

**Aam013071**	353	0.20	0.37	0.16	0.80	7.58	1.02	254.92
	607	0.34	0.87	0.53	0.82	8.47	1.01	282.18
**Aam013411**	NO							
**Aam013450**	130	0.75	2.46	1.71	0.84	13.60	1.00	1289.13
	483	0.54	2.20	1.66	0.89	22.05	1.00	1859.83
	525	0.79	2.69	1.89	0.83	12.45	1.00	1209.43
	776	0.58	1.92	1.34	0.84	14.11	1.00	1405.38
**Aam013663**	389	1.14	4.37	3.23	0.84	28.14	1.01	399.69
	390	0.93	3.55	2.61	0.83	27.87	1.01	407.52
**Aam014899**	NO							
**Aam017081**	28	0.20	0.41	0.20	0.84	8.50	1.00	2177.50
	30	0.39	1.55	1.16	0.90	15.02	1.00	1098.06
	52	0.31	1.02	0.71	0.87	10.74	1.00	1307.44
	53	0.25	0.64	0.40	0.83	7.65	1.00	1964.69
	57	0.80	3.13	2.34	0.85	9.37	1.00	1186.96
**Aam019908**	NO							
**Aam022194**	123	0.93	3.59	2.66	0.83	10.75	1.00	1981.61
	261	0.56	2.34	1.78	0.85	12.47	1.00	1241.04
**Aam038631**	NO							
**Aam044051**	10	0.61	1.87	1.26	0.81	8.65	1.00	1177.56
	134	0.72	2.50	1.79	0.82	8.86	1.00	1116.57
	135	0.80	2.71	1.91	0.81	8.29	1.00	935.48
	150	0.15	0.43	0.28	0.81	8.54	1.00	639.48
	164	0.95	3.59	2.64	0.83	10.03	1.00	694.96
**Aam044297**	111	0.36	0.98	0.62	0.81	10.01	1.00	913.50
	181	0.67	2.22	1.54	0.83	11.10	1.00	837.80
	471	0.72	2.26	1.54	0.80	9.38	1.00	843.56
**Aam044505**	NO							
**Aam044597**	23	0.43	1.69	1.27	0.85	13.46	1.00	909.98
	519	0.63	1.94	1.32	0.80	9.74	1.00	1222.03
**Aam044860**	NO							
**Aam045646**	32	0.84	2.47	1.63	0.81	13.12	1.00	1013.8

								3
**Aam045648**	256	0.12	0.28	0.15	0.85	18.45	1.00	1804.06
**Aam045742**	NO							
**Aam045814**	593	0.79	2.75	1.96	0.81	13.12	1.00	620.21
**Aam045853**	NO							
**Aam045861**	1046	0.12	0.41	0.30	0.86	19.12	1.00	1052.83
**Aam046130**	NO							
**Aam046915**	27	0.68	2.11	1.43	0.81	6.31	1.00	624.31
	32	0.91	2.98	2.07	0.82	6.72	1.00	793.98
	141	0.26	0.52	0.27	0.81	6.38	1.01	383.88
	181	0.46	1.48	1.02	0.84	8.00	1.01	444.60
**Aam048341**	124	0.82	2.86	2.04	0.83	10.38	1.00	1136.58
	219	0.82	2.76	1.93	0.82	9.86	1.00	1219.48
**Aam051597**	73	0.91	2.87	1.96	0.80	6.87	1.00	639.98
**Aam052293**	207	0.76	2.47	1.71	0.81	7.27	1.00	739.20
**Aam055616**	380	0.46	1.28	0.83	0.81	7.48	1.00	559.56
**Aam055741**	NO							
**Aam056318**	710	0.36	1.37	1.00	0.85	14.33	1.00	659.85
	960	0.92	3.15	2.23	0.81	10.85	1.00	631.09
**Aam063676**	NO							
**Aam074009**	8	0.88	3.35	2.47	0.85	12.91	1.00	588.27
	43	1.06	3.78	2.72	0.82	10.41	1.00	558.94
	50	0.27	1.25	0.98	0.91	22.97	1.00	1006.10
	55	0.54	2.28	1.74	0.88	16.53	1.00	826.05
	57	0.49	1.58	1.09	0.85	13.64	1.00	1016.07
	58	0.72	3.14	2.42	0.87	15.54	1.00	642.07
**Aam075912**	7	0.76	2.36	1.60	0.82	9.36	1.00	2626.72
**Aam076244**	NO							
**Aam076551**	9	0.61	1.80	1.18	0.82	10.76	1.00	535.93
**Aam077266**	243	0.21	0.53	0.32	0.83	19.62	1.00	761.28
**Aam078199**	221	0.86	3.10	2.24	0.85	16.96	1.00	728.26
	457	0.57	1.65	1.07	0.84	16.37	1.00	1010.37
	478	1.12	3.85	2.73	0.82	14.42	1.00	848.17
**Aam078452**	29	0.77	2.66	1.88	0.82	14.40	1.00	623.45
**Aam079585**	266	0.72	2.22	1.50	0.81	8.09	1.00	995.15
	321	0.70	2.41	1.71	0.84	10.25	1.00	1040.6

								6
**Aam079965**	164	0.62	1.89	1.27	0.81	7.02	1.00	819.12
	175	0.65	1.91	1.26	0.81	6.77	1.00	748.94
	176	0.64	1.63	1.00	0.80	6.63	1.00	829.72
	238	0.95	3.24	2.29	0.80	6.53	1.00	696.07
	246	1.02	3.56	2.54	0.81	7.15	1.00	686.44
	264	0.64	2.38	1.75	0.86	10.11	1.00	778.06
	265	0.39	1.32	0.93	0.86	10.02	1.00	740.61
	303	0.43	1.60	1.17	0.86	10.20	1.00	882.03
	305	0.28	0.68	0.41	0.82	7.29	1.00	663.79
	331	0.24	0.63	0.39	0.84	8.76	1.00	643.08
**Aam081307**	2	0.20	0.50	0.30	0.81	13.37	1.00	755.29
	12	0.10	0.22	0.11	0.81	12.89	1.00	567.95
	75	1.10	3.59	2.48	0.80	12.46	1.00	817.15
	84	0.70	2.32	1.63	0.81	13.01	1.00	844.97
**Aam081496**	19	0.51	2.02	1.51	0.83	16.11	1.00	953.62
	25	0.54	1.61	1.06	0.81	14.66	1.00	847.66
	558	0.70	2.48	1.79	0.84	17.30	1.00	1105.49
	936	0.89	3.19	2.30	0.83	15.94	1.00	1174.83
**Aam081889**	NO							
**Aam082363**	NO							
**Aam082779**	224	0.64	2.52	1.88	0.86	18.81	1.00	1052.90
	225	0.99	3.50	2.50	0.82	13.16	1.00	1148.16
	228	0.70	2.29	1.59	0.82	13.32	1.00	904.02
	346	0.77	2.35	1.57	0.81	12.34	1.00	852.87
	351	0.48	1.65	1.17	0.86	18.28	1.00	739.98
	638	0.61	1.90	1.30	0.81	12.66	1.00	826.16
	733	0.94	3.19	2.25	0.82	13.19	1.00	1123.71
	990	0.74	2.29	1.55	0.81	12.64	1.00	855.01
**Aam083292**	NO							
**Aam083376**	170	0.12	0.26	0.14	0.85	11.26	1.01	428.38
	543	0.67	2.01	1.33	0.80	7.85	1.00	1174.67
	550	0.67	1.96	1.29	0.81	8.24	1.00	1166.15
**Aam083451**	212	0.77	3.13	2.36	0.87	15.36	1.00	1790.42
	231	0.52	2.04	1.51	0.87	15.13	1.00	823.62
	441	1.04	3.46	2.43	0.82	10.68	1.00	1963.8

								9
	445	0.90	3.45	2.55	0.84	12.21	1.00	1722.02
**Aam083978**	632	0.86	2.83	1.97	0.80	13.08	1.00	1116.05
**Aam084654**	NO							
**Aam085207**	560	1.16	4.15	3.00	0.82	15.39	1.00	705.90
	586	0.90	3.25	2.35	0.81	14.45	1.00	778.44
	593	0.84	3.23	2.39	0.82	15.95	1.00	760.54
	838	1.13	3.97	2.84	0.80	14.18	1.00	707.00
**Aam085507**	12	0.57	1.66	1.09	0.81	9.08	1.00	601.06
	87	0.60	2.07	1.47	0.83	10.43	1.00	641.26
	113	0.78	2.72	1.94	0.83	10.54	1.00	665.53
	178	1.10	3.85	2.75	0.81	8.93	1.00	937.77
	249	0.71	2.55	1.84	0.84	11.33	1.00	648.68
	260	1.00	3.44	2.44	0.82	9.98	1.00	900.21
	261	1.12	3.87	2.75	0.81	8.94	1.00	913.92
	365	0.74	2.69	1.95	0.84	11.60	1.00	744.92
**Aam085920**	257	0.97	3.38	2.41	0.82	18.20	1.01	325.84
	301	0.43	1.54	1.11	0.87	27.60	1.01	456.49
	353	0.49	1.41	0.92	0.83	18.88	1.01	498.77
	371	0.49	1.69	1.19	0.88	29.45	1.01	405.66
**Aam085954**	28	0.21	0.75	0.53	0.88	16.04	1.00	521.35
	470	0.69	2.11	1.42	0.81	9.08	1.00	932.80
	663	0.59	1.90	1.31	0.84	10.96	1.00	804.29
	741	0.94	3.03	2.08	0.81	8.85	1.00	1222.65
**Aam086219**	341	0.43	1.32	0.90	0.82	8.73	1.00	819.18
**Aam086287**	155	0.31	0.96	0.65	0.84	11.29	1.00	811.19
	451	0.09	0.13	0.04	0.80	8.72	1.00	2211.15
**Aam086482**	319	0.73	2.22	1.49	0.80	11.87	1.00	554.65
	984	0.97	3.34	2.37	0.81	12.85	1.00	578.34
**Aam086603**	30	0.21	0.51	0.30	0.80	8.98	1.00	660.96
	203	0.28	0.65	0.37	0.81	9.33	1.00	656.68
	363	0.38	1.06	0.69	0.83	10.68	1.00	770.38
	508	0.48	1.60	1.12	0.86	13.17	1.00	794.22
	521	0.87	2.89	2.02	0.81	9.05	1.00	969.07
**Aam087015**	NO							
**Aam087017**	27	0.15	0.36	0.21	0.87	24.78	1.00	817.43
	35	0.69	2.32	1.64	0.82	15.86	1.00	680.54
	41	0.50	2.18	1.68	0.90	33.68	1.00	508.57
	45	0.20	0.43	0.23	0.81	14.91	1.00	1086.54

	49	0.17	0.46	0.29	0.81	15.64	1.00	1089.58
	221	0.87	3.10	2.24	0.82	16.10	1.00	709.12
	453	0.33	1.21	0.88	0.87	23.97	1.00	741.87
	572	0.19	0.62	0.42	0.86	21.10	1.00	1012.03
**Aam087037**	NO							
**Aam087428**	50	0.20	0.87	0.67	0.89	23.71	1.00	825.75
**Aam087461**	201	0.24	0.56	0.32	0.83	10.21	1.01	369.99
	525	0.10	0.20	0.10	0.82	9.52	1.01	336.76
**Aam087490**	NO							
**Aam087513**	NO							
**Aam087837**	2250	0.84	2.98	2.14	0.81	15.37	1.00	669.13
	2251	0.56	1.89	1.33	0.82	16.03	1.00	748.36
**Aam087989**	481	0.36	0.79	0.43	0.82	8.83	1.00	725.67
**Aam088035**	NO							
**Aam088045**	254	0.70	2.46	1.76	0.84	8.47	1.01	386.46
	280	0.45	1.36	0.91	0.83	7.94	1.01	306.59
	332	0.76	2.76	2.00	0.84	8.95	1.01	383.99
**Aam088209**	111	1.07	3.59	2.52	0.80	9.75	1.00	702.43
**Aam088351**	NO							
**Aam088368**	NO							
**Aam088382**	528	0.57	2.06	1.49	0.83	23.51	1.00	633.41
	1855	1.01	3.93	2.92	0.84	24.59	1.01	462.03
**Aam322360**	86	0.76	2.21	1.45	0.82	9.02	1.00	1795.84
	186	0.71	2.58	1.87	0.84	10.20	1.00	1451.17
	206	1.02	3.58	2.56	0.82	9.12	1.00	1180.30
	220	0.86	2.99	2.13	0.82	9.37	1.00	1576.32
**Aam324992**	12	0.97	3.52	2.55	0.82	7.97	1.00	959.41
	16	0.23	0.60	0.38	0.84	9.38	1.00	537.60
	44	0.79	2.53	1.74	0.81	7.46	1.00	1013.66
	90	0.84	2.89	2.05	0.83	8.46	1.00	757.72
**Aam325436**	101	0.99	3.39	2.40	0.80	11.09	1.01	427.63
	174	0.20	0.58	0.38	0.85	15.18	1.01	419.79
	557	0.78	2.72	1.94	0.81	11.99	1.01	411.57
	669	0.75	2.57	1.82	0.82	12.50	1.01	408.06
	793	0.63	2.27	1.64	0.84	13.98	1.01	379.95
**Aam327516**	4	0.11	0.21	0.10	0.85	8.61	1.00	1957.29

	10	1.00	3.30	2.29	0.81	6.79	1.00	1183.03
	20	0.95	3.39	2.44	0.82	7.11	1.00	1319.82
	41	0.44	1.70	1.26	0.88	11.05	1.00	779.99
	44	0.22	0.38	0.16	0.80	6.20	1.00	850.07
	45	0.12	0.29	0.17	0.85	8.69	1.00	1415.29
	46	0.17	0.52	0.34	0.84	7.96	1.00	1122.83
	92	0.80	2.70	1.90	0.84	7.94	1.00	1154.76
**Aam332970**	78	0.28	0.60	0.32	0.82	8.38	1.00	1924.97
**Aam349697**	83	0.23	0.79	0.56	0.87	31.33	1.00	1412.89
	88	0.17	0.43	0.26	0.83	21.72	1.00	1746.00
	475	0.86	3.18	2.32	0.85	25.24	1.00	768.33
**Aam357923**	119	0.67	2.22	1.55	0.81	17.78	1.00	1468.64
