# Supplementary material for: Diel rewiring and positive selection of ancient plant proteins enabled evolution of CAM photosynthesis in Agave
Source: BMC Genomics. 2018 Aug 6;19:588. doi: 10.1186/s12864-018-4964-7 (PMC6090859; doi:10.1186/s12864-018-4964-7)
Supplement: Supplementary file 13 — Figure S3. Positive selection region in chloroplast β-amylase (CT-BMY). (PDF 171 kb) [file 12864_2018_4964_MOESM13_ESM.pdf]

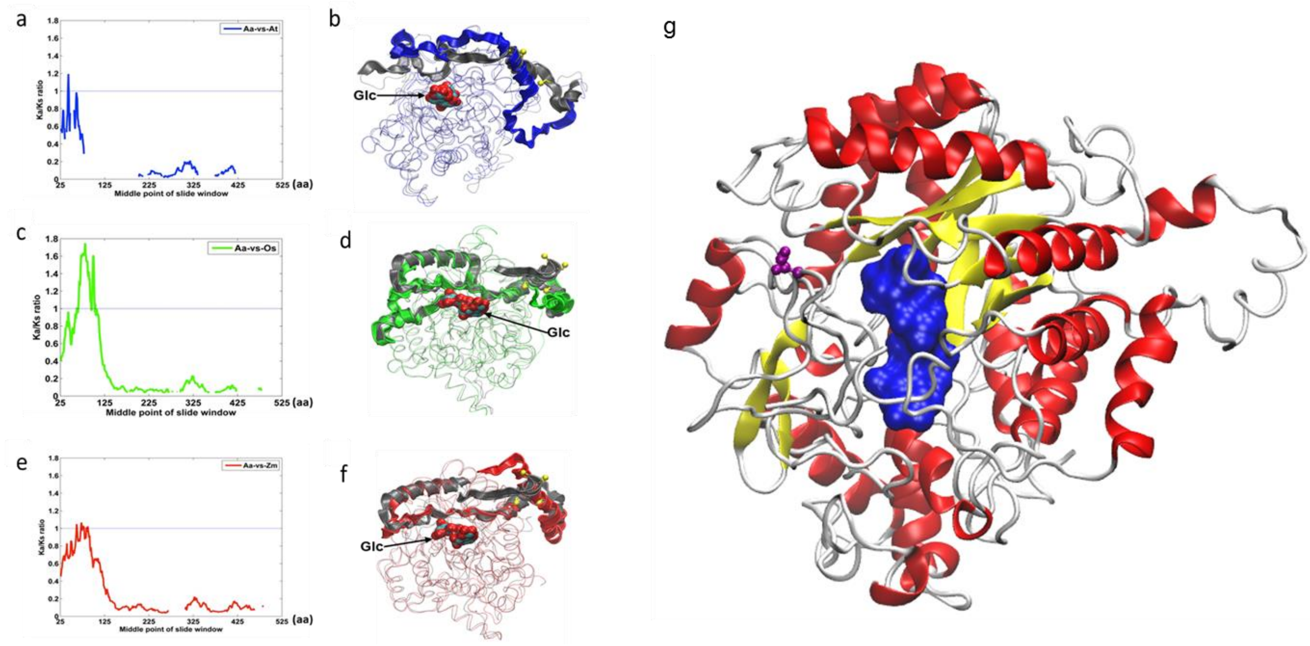

Figure S3. Positive selection region in chloroplast β-amylase (CT-BMY). (**a**) Ka/Ks profile of *Agave americana* (Aa) versus *Arabidopsis thaliana* (At); (**b**) superimposed structures in Aa and At, with the positive selection region highlighted; (**c**) Ka/Ks profile of Aa versus *Oryza sativa* (Os); (**d**) superimposed structures in Aa and Os, with the positive selection region highlighted; (**e**) Ka/Ks profile of Aa versus *Zea may*s (Zm); (**f**) superimposed structures in Aa and Zm, with the positive selection region highlighted. (**g**) The structure model of CT-BMY (Aam049326). This model indicates that the beta-amylase (helices in red, strands in yellow and coils in white) may react with glucose or maltose. A D-glucose substrate (Glc, blue surface) is bound at the active site in the model. E24 (codon 73, purple spheres and sticks) is located in a mobile loop region that may act as a cover of the pocket that holds the substrate. A glucose (Glc) substrate that may bind to the Aa CT-BMY is marked by an arrow. The proteins are colored in grey for Aa, blue for At, green for Os and red for Zm.
