# Supplementary material for: Diel rewiring and positive selection of ancient plant proteins enabled evolution of CAM photosynthesis in Agave
Source: BMC Genomics. 2018 Aug 6;19:588. doi: 10.1186/s12864-018-4964-7 (PMC6090859; doi:10.1186/s12864-018-4964-7)
Supplement: Supplementary file 14 — Table S11. List of Agave americana genes with morning-to-night shift in expression pattern as compared with the orthologous genes in Arabidopsis. (PDF 70 kb) [file 12864_2018_4964_MOESM14_ESM.pdf]

Table S11. List of *Agave americana* genes with morning-to-night shift in expression pattern as compared with the orthologous genes in *Arabidopsis*.

Agave gene	*Arabidopsis* gene	Function category	Definition	Ortholog clade
Aam073900	AT5G05270	Anthocyanin biosynthetic process	Chalcone-flavanone isomerase family protein	NVP:C3:CAM:C4
Aam003740	AT5G54130	Calcium binding	Calcium-binding endonuclease/exonuclease/phosphatase	NVP:C3:CAM:C4
Aam022373	AT5G17300	Circadian-output	Reveille 1 (rve1)	C3:CAM:C4
Aam340116	AT5G64460	Cytokinin pathway	Phosphoglycerate mutase family protein	NVP:C3:CAM:C4
Aam013303	AT1G10740	Glycerol biosynthetic process	Alpha/beta-Hydrolases superfamily protein	NVP:C3:CAM:C4
Aam086075	AT1G44446	Photosynthesis-chlorophyll biosynthesis	Pheophorbide a oxygenase family protein	NVP:C3:CAM:C4
Aam010047	AT1G64770	Photosynthesis-PET	NDH-dependent cyclic electron flow 1; NDF2	C3:CAM:C4
Aam084849	AT4G29950	RAB gtpase activator	Ypt/Rab-GAP domain of gyp1p superfamily protein	NVP:C3:CAM:C4
Aam291140	AT5G18600	Redox homeostasis	Thioredoxin superfamily protein	NVP:C3:CAM:C4
Aam027379	AT3G50980	Stress responsive	Dehydrin xero 1 (XERO1)	C3:CAM
Aam054885	AT5G42760	Stress responsive	Leucine carboxyl methyltransferase	NVP:C3:CAM:C4
Aam011975	AT1G75500	Transporter	Walls are thin 1 (wat1)	NVP:C3:CAM:C4
Aam043718	AT5G47560	Transporter (malate)	Tonoplast dicarboxylate transporter (TDT)	NVP:C3:CAM:C4
Aam018832	AT4G22200	Transporter (Stomatal movement)	Potassium transport 2/3	NVP:C3:CAM:C4
Aam003354	AT2G15020	Unknown	Unknown protein	C3:CAM:C4
Aam004076	AT4G24700	Unknown	Unknown protein	C3:CAM:C4
Aam017052	AT3G15810	Unknown	Protein of unknown function (DUF567)	C3:CAM:C4
Aam048214	AT1G21000	Unknown	PLATZ transcription factor family protein	NVP:C3:CAM:C4
Aam086795	AT1G22930	Unknown	T-complex protein 11	NVP:C3:CAM:C4
Aam313665	AT5G55570	Unknown	Unknown protein	NVP:C3:CAM:C4
Aam328864	AT3G29240	Unknown	Protein of unknown function (DUF179)	NVP:C3:CAM:C4
Aam336929	AT1G15230	Unknown	Unknown protein	NVP:C3:CAM
