# Supplementary material for: Diel rewiring and positive selection of ancient plant proteins enabled evolution of CAM photosynthesis in Agave
Source: BMC Genomics. 2018 Aug 6;19:588. doi: 10.1186/s12864-018-4964-7 (PMC6090859; doi:10.1186/s12864-018-4964-7)
Supplement: Supplementary file 15 — Table S12. List of Agave americana genes with afternoon-to-night shift in expression pattern as compared with the orthologous genes in Arabidopsis. (PDF 69 kb) [file 12864_2018_4964_MOESM15_ESM.pdf]

Table S12. List of *Agave americana* genes with afternoon-to-night shift in expression pattern as compared with the orthologous genes in *Arabidopsis*.

Agave gene	*Arabidopsis* gene	Function category	Definition	Ortholog clade
Aam007668	AT2G38050	Brassinosteroid pathway	3-oxo-5-alpha-steroid 4-dehydrogenase; DE-ETIOLATED 2	NVP:C3:CAM:C4
Aam043341	AT4G24210	Gibberellin pathway	F-box family protein; SLEEPY1 (SLY1)	NVP:C3:CAM:C4
Aam328824	AT1G47830	Intracellular protein transport	SNARE-like superfamily protein	NVP:C3:CAM:C4
Aam008820	AT2G02400	Lignin biosynthesis	NAD(P)-binding Rossmann-fold superfamily protein	C3:CAM
Aam007892	AT1G62040	Phosphatidylinositol biosynthesis	Ubiquitin-like superfamily protein	NVP:C3:CAM:C4
Aam014937	AT1G54210	Phosphatidylinositol biosynthesis	Ubiquitin-like superfamily protein	NVP:C3:CAM:C4
Aam049417	AT3G61470	Photosynthesis - antenna	Photosystem I light harvesting complex gene 2 (LHCA2)	NVP:C3:CAM:C4
Aam015317	AT1G08380	Photosynthessi-PET	Photosystem I subunit O	NVP:C3:CAM:C4
Aam008410	AT4G21105	Redox homeostasis	Cytochrome-c oxidases;electron carriers	C3:CAM:C4
Aam019477	AT5G60860	Small gtpase	RAB gtpase homolog A1F (RABA1f)	NVP:C3:CAM:C4
Aam076638	AT5G47120	Stress responsive	BAX inhibitor 1 (BI1)	NVP:C3:CAM:C4
Aam309984	AT2G46330	Sugar metabolic pathway	Arabinogalactan protein 16 (AGP16)	NVP:C3:CAM:C4
Aam312763	AT5G14910	Transporter	Heavy metal transport/detoxification superfamily protein	NVP:C3:CAM:C4
Aam012414	AT1G56260	Unknown	Unknown protein	NVP:C3:CAM:C4
Aam016072	AT5G37480	Unknown	Unknown protein	NVP:C3:CAM:C4
Aam017752	AT1G72170	Unknown	Domain of unknown function (duf543)	NVP:C3:CAM:C4
Aam047120	AT4G20150	Unknown	Unknown protein	NVP:C3:CAM:C4
Aam073509	AT1G65900	Unknown	Unknown protein	NVP:C3:CAM:C4
Aam287556	AT5G63905	Unknown	Unknown protein	C3:CAM:C4
Aam321654	AT1G27290	Unknown	Unknown protein	C3:CAM:C4
