# Supplementary material for: Diel rewiring and positive selection of ancient plant proteins enabled evolution of CAM photosynthesis in Agave
Source: BMC Genomics. 2018 Aug 6;19:588. doi: 10.1186/s12864-018-4964-7 (PMC6090859; doi:10.1186/s12864-018-4964-7)
Supplement: Supplementary file 16 — Figure S4. Phylogenetic trees and diel gene expression patterns of multi-gene ortholog groups (i.e., with a total of more than two Agave and Arabidopsis genes) listed Fig. 4a. (PDF 164 kb) [file 12864_2018_4964_MOESM16_ESM.pdf]

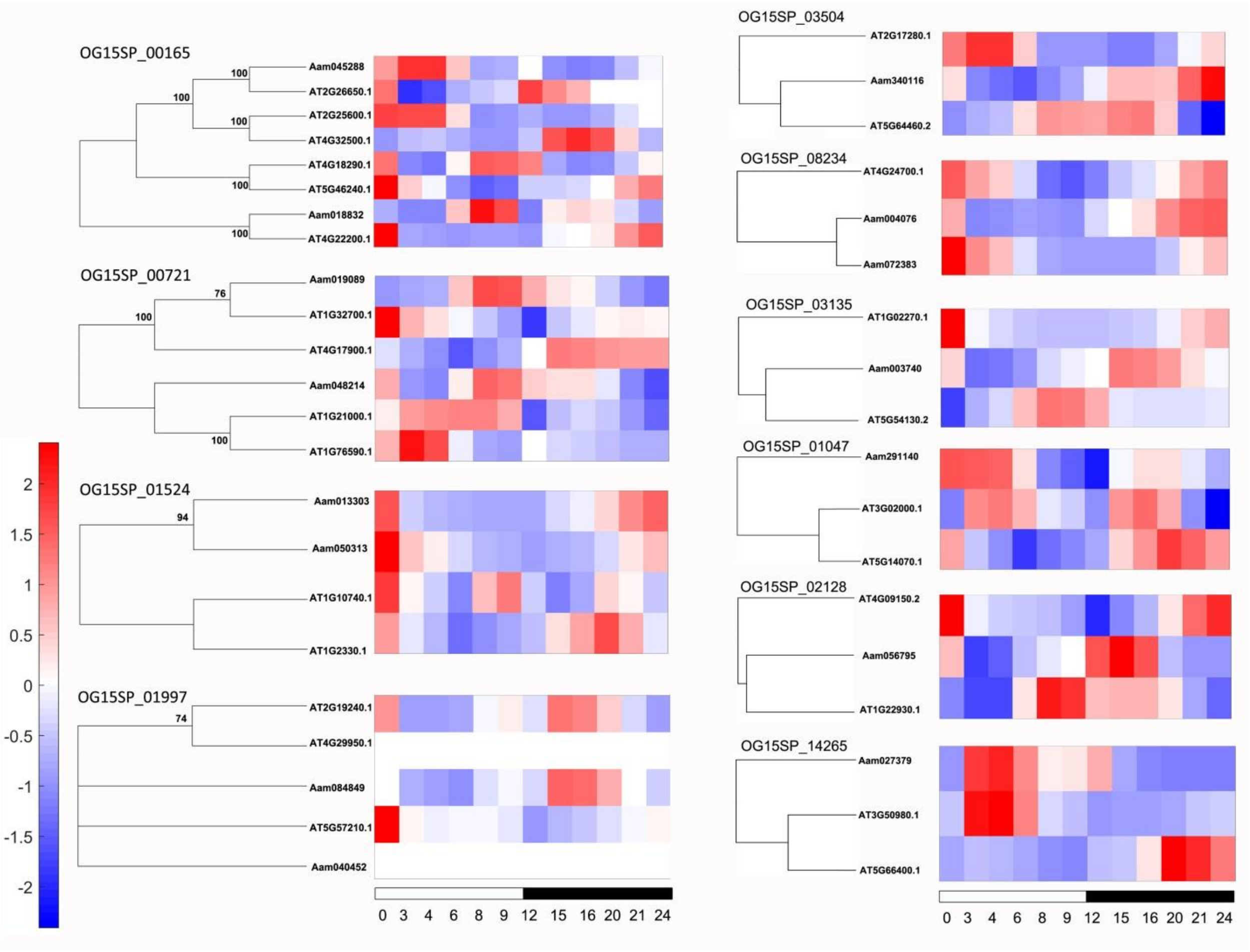

Figure S4. Phylogenetic trees and diel gene expression patterns of multi-gene ortholog groups (i.e., with a total of more than two *Agave* and *Arabidopsis* genes) listed Figure 4a. The z-score normalized expression patterns were plotted by heatmaps. The blank rows indicate no gene expression detected by transcriptome-sequencing. White and black bars indicate daytime (12-hour) and nighttime (12-hour), respectively.
