# Supplementary material for: Diel rewiring and positive selection of ancient plant proteins enabled evolution of CAM photosynthesis in Agave
Source: BMC Genomics. 2018 Aug 6;19:588. doi: 10.1186/s12864-018-4964-7 (PMC6090859; doi:10.1186/s12864-018-4964-7)
Supplement: Supplementary file 18 — Table S13. List of circadian clock genes in Agave americana and Arabidopsis thaliana. (PDF 18 kb) [file 12864_2018_4964_MOESM18_ESM.pdf]

Table S13. List of circadian clock genes in *Agave americana* and *Arabidopsis thaliana*.

Category	*Arabidopsis* gene name	Description	*Agave* gene ID
Core	CHE	CCA1 HIKING EXPEDITION (CHE)	Aam080997
Core	APRR5	PSEUDO-RESPONSE REGULATOR 5	Aam054111
Core	APRR7	PSEUDO-RESPONSE REGULATOR 7	Aam087030
Core	CCA1	CIRCADIAN CLOCK ASSOCIATED 1	Aam006353
Core	PCL1; LUX	PHYTOCLOCK 1	Aam048752
Core	GI	GIGANTEA	Aam087254
Core	TOC1	TIMING OF CAB EXPRESSION 1	Aam086245
Core	ZTL	ZEITLUPE	Aam013645
Input	cry1	CRYPTOCHROME 1	Aam045607
Input	cry2	CRYPTOCHROME 2	Aam348626
Input	ELF3	EARLY FLOWERING 3	Aam085988
Input	Phot2	PHOTOTROPIN 2	Aam086385
Input	phyB	PHYTOCHROME B	Aam076417
Input	PIF3	PHYTOCHROME INTERACTING FACTOR 3	Aam087593
Input	FKF1	FLAVIN-BINDING, KELCH REPEAT, F BOX 1	Aam009867
Output	CCR2	CAROTENOID AND CHLOROPLAST REGULATION 2	Aam342422
Output	RVE1	REVEILLE 1	Aam022373
Output	AMY3	ALPHA-AMYLASE-LIKE 3	Aam054669
Output	DPE1	DISPROPORTIONATING ENZYME 1	Aam044194
Output	PORA	PROTOCHLOROPHYLLIDE OXIDOREDUCTASE A	Aam010547
Output	GWD1	STARCH EXCESS 1	Aam003444
Output	CDF2	CYCLING DOF FACTOR 2	Aam010085
