# Supplementary material for: Diel rewiring and positive selection of ancient plant proteins enabled evolution of CAM photosynthesis in Agave
Source: BMC Genomics. 2018 Aug 6;19:588. doi: 10.1186/s12864-018-4964-7 (PMC6090859; doi:10.1186/s12864-018-4964-7)
Supplement: Supplementary file 19 — Figure S6. Intrinsically disordered protein regions (IDPRs) and structural models of the circadian clock proteins in Agave americana. (PDF 153 kb) [file 12864_2018_4964_MOESM19_ESM.pdf]

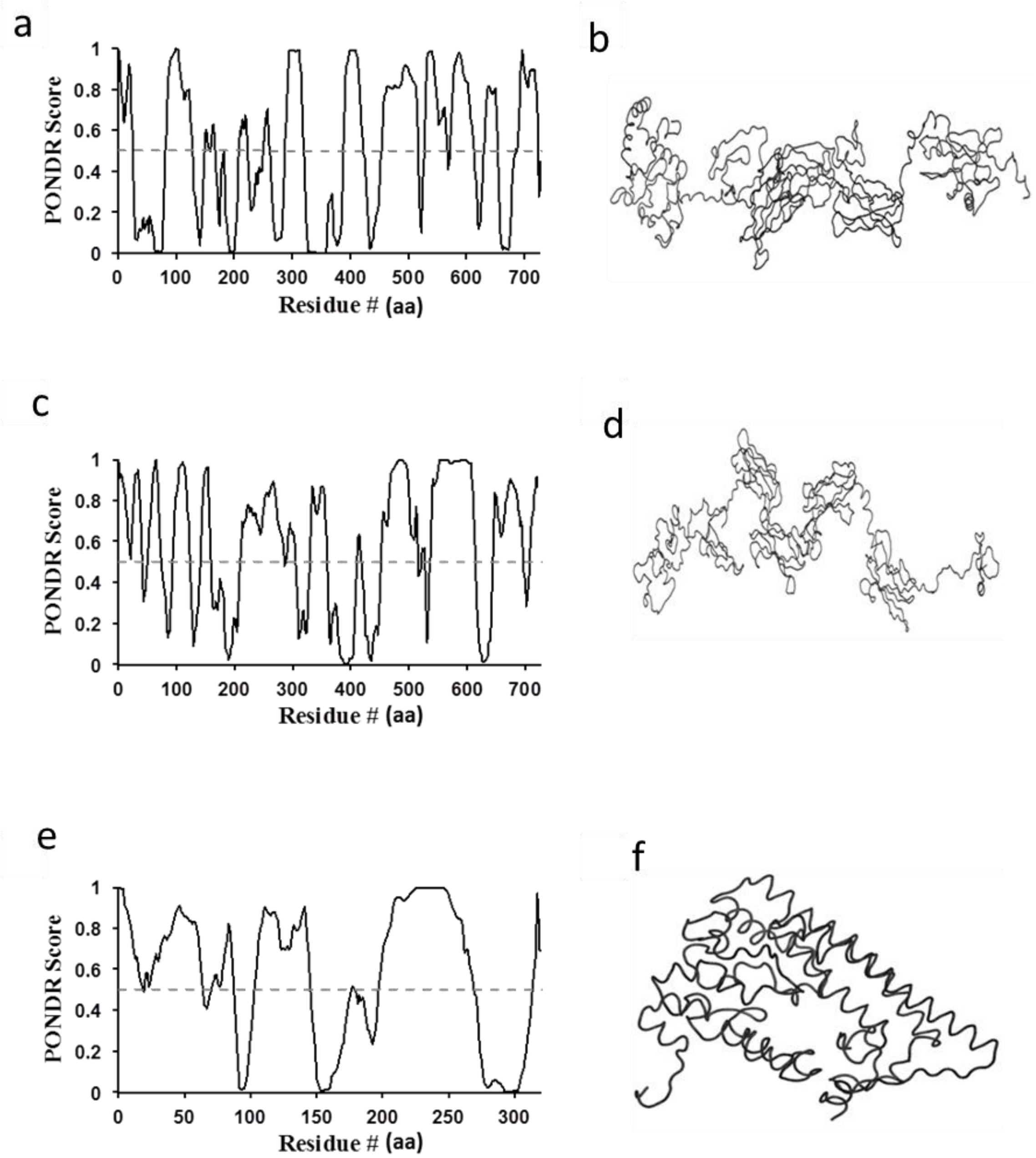

Figure S6. Intrinsically disordered protein regions (IDPRs) and structural models of the circadian clock proteins in *Agave americana*. (**a**) PONDR-score profile of LATE ELONGATED HYPOCOTYL (LHY, Aam006353); (**b**) structural model of LHY; (**c**) PONDR-score profile of EARLY FLOWERING 3 (ELF3, Aam085988), (**d**) structural model of ELF3, (**e**) PONDR-score profile of LUX ARRHYTHMO (LUX, Aam048752), and (**f**) structural model of LUX.
