# Supplementary material for: Diel rewiring and positive selection of ancient plant proteins enabled evolution of CAM photosynthesis in Agave
Source: BMC Genomics. 2018 Aug 6;19:588. doi: 10.1186/s12864-018-4964-7 (PMC6090859; doi:10.1186/s12864-018-4964-7)
Supplement: Supplementary file 20 — Table S14. Intrinsically disordered protein region (IDPR) in protein sequences under positive selection. (PDF 33 kb) [file 12864_2018_4964_MOESM20_ESM.pdf]

Table S14. Intrinsically disordered protein region (IDPR) in protein sequences under positive selection.

Gene ID	Gene name	Protein sequence length (aa)	Longest IDPR	R_{dis}*	S_{PONDR}**
Aam088315	GAUT7	654	N113-E173	0.200	0.277
Aam049326	CT-BMY	544	N21-N40	0.193	0.294
Aam082045	SS2	646	V57-Q150	0.361	0.343
Aam048341	PPCK1	280	P237-A257	0.186	0.290
Aam051010	PPDK-RP	421	N87-A153	0.508	0.497
Aam002877	PsaD2	244	D83-G118	0.385	0.412
Aam085988	ELF3	720	G536-N613	0.638	0.589
Aam048752	LUX	320	M197-V268	0.634	0.576
Aam006353	LHY	727	V453-G516	0.534	0.513

*Ratio of disordered residues.

**The average PONDR VL-XT score of the entire protein.
