# Supplementary material for: Correction to: Diel rewiring and positive selection of ancient plant proteins enabled evolution of CAM photosynthesis in Agave
Source: BMC Genomics. 2019 Apr 10;20:279. doi: 10.1186/s12864-019-5663-8 (PMC6456932; doi:10.1186/s12864-019-5663-8)
Supplement: Supplementary file 1 — Table S1. Percentage of the gene set in each individual species distributed into different ortholog clades. (DOCX 20 kb) [file 12864_2019_5663_MOESM1_ESM.docx]

**Additional file 2**: **Table S1**. Percentage of the gene set in each individual species distributed into different ortholog clades.

| Ortholog |  | NVP |  |  | C_3_ monocot | |  |  | C_3_ dicot |  |  | CAM |  |  | C_4_ |  |
| --- | --- | --- | --- | --- | --- | --- | --- | --- | --- | --- | --- | --- | --- | --- | --- | --- |
| clade | Cre | Ppa | Smo | Mac | Bdi | Osa | | Stu | Ptr | Ath | Aam | Ade | Ate | Sit | Sbi | Zma |
| NVP-only | **46** | **27** | **41** | 0 | 0 | 0 | | 0 | 0 | 0 | 0 | 0 | 0 | 0 | 0 | 0 |
| C_3_-only | 0 | 0 | 0 | 7 | 4 | 13 | | **34** | **19** | **20** | 0 | 0 | 0 | 0 | 0 | 0 |
| CAM-only | 0 | 0 | 0 | 0 | 0 | 0 | | 0 | 0 | 0 | 18 | 17 | 19 | 0 | 0 | 0 |
| C_4_-only | 0 | 0 | 0 | 0 | 0 | 0 | | 0 | 0 | 0 | 0 | 0 | 0 | 8 | 6 | 17 |
| NVP:C_3_:CAM:C_4_ | **47** | **66** | **53** | **61** | **53** | **44** | | **37** | **52** | **54** | **56** | **56** | **53** | **48** | **51** | **47** |
| NVP:C_3_:CAM | 1 | 2 | 1 | 1 | 0 | 0 | | 1 | 2 | 1 | 1 | 1 | 2 | 0 | 0 | 0 |
| NVP:CAM:C_4_ | 0 | 0 | 0 | 0 | 0 | 0 | | 0 | 0 | 0 | 0 | 0 | 0 | 0 | 0 | 0 |
| NVP:CAM | 2 | 2 | 1 | 0 | 0 | 0 | | 0 | 0 | 0 | 1 | 1 | 1 | 0 | 0 | 0 |
| C_3_:CAM:C_4_ | 0 | 0 | 0 | **23** | **19** | **17** | | **14** | **18** | **17** | **19** | **20** | **20** | **18** | **19** | **16** |
| C_3_:CAM | 0 | 0 | 0 | 4 | 0 | 0 | | 10 | 5 | 3 | 5 | 5 | 5 | 0 | 0 | 0 |
| CAM:C_4_ | 0 | 0 | 0 | 0 | 0 | 0 | | 0 | 0 | 0 | 0 | 0 | 0 | 0 | 0 | 0 |
| C_3_:C_4_ | 0 | 0 | 0 | **3** | **22** | **24** | | 2 | 2 | 3 | 0 | 0 | 0 | **25** | **23** | **18** |
| NVP:C_3_:C_4_ | 1 | 1 | 2 | 1 | 1 | 1 | | 2 | 1 | 1 | 0 | 0 | 0 | 1 | 1 | 1 |
| NVP:C_3_ | 2 | 2 | 1 | 0 | 0 | 0 | | 1 | 1 | 1 | 0 | 0 | 0 | 0 | 0 | 0 |
| NVP:C_4_ | 0 | 0 | 0 | 0 | 0 | 0 | | 0 | 0 | 0 | 0 | 0 | 0 | 0 | 0 | 0 |

Note: NVP-only, C3-only, CAM-only and C4-only represent orthologs specific to NVP (i.e. non-vascular plants), C_3_, CAM and C_4_ species, respectively. NVP:C_3_:CAM:C_4_ represents orthologs shared by NVP, C_3_, CAM and C_4_. NVP:C_3_:CAM represents orthologs shared only by NVP, C_3_ and CAM. NVP:CAM:C_4_ represents orthologs shared only by NVP, CAM and C_4_. NVP:CAM represents orthologs shared only by NVP and CAM. C_3_:CAM:C_4_ represents orthologs shared only by C_3_, CAM and C_4_. C_3_:CAM represents orthologs shared only by C_3_ and CAM. CAM:C_4_ represents orthologs shared only by CAM and C_4_. C_3_:C_4_ represents orthologs shared only by C_3_ and C_4_. NVP:C_3_:C_4_ represents orthologs shared only by NVP, C_3_ and C_4_. NVP:C_3_ represents orthologs shared only by NVP and C_3_. NVP:C_4_ represents orthologs shared only by NVP and C_4_.
